# ASTRAL-Pro: Quartet-Based Species-Tree Inference despite Paralogy

**DOI:** 10.1093/molbev/msaa139

**Published:** 2020-09-04

**Authors:** Chao Zhang, Celine Scornavacca, Erin K Molloy, Siavash Mirarab

**Affiliations:** 1 Bioinformatics and Systems Biology, University of California San Diego, San Diego, CA; 2 ISEM, Université de Montpellier, CNRS, IRD, EPHE, Montpellier, France; 3 Department of Computer Science, University of Illinois at Urbana-Champaign, Champaign, IL; 4 Department of Electrical and Computer Engineering, University of California San Diego, San Diego, CA

**Keywords:** species-tree inference, gene duplication and loss, incomplete lineage sorting

## Abstract

Phylogenetic inference from genome-wide data (phylogenomics) has revolutionized the study of evolution because it enables accounting for discordance among evolutionary histories across the genome. To this end, summary methods have been developed to allow accurate and scalable inference of species trees from gene trees. However, most of these methods, including the widely used ASTRAL, can only handle single-copy gene trees and do not attempt to model gene duplication and gene loss. As a result, most phylogenomic studies have focused on single-copy genes and have discarded large parts of the data. Here, we first propose a measure of quartet similarity between single-copy and multicopy trees that accounts for orthology and paralogy. We then introduce a method called ASTRAL-Pro (ASTRAL for PaRalogs and Orthologs) to find the species tree that optimizes our quartet similarity measure using dynamic programing. By studying its performance on an extensive collection of simulated data sets and on real data sets, we show that ASTRAL-Pro is more accurate than alternative methods.

## Introduction

The evolutionary history of a gene can differ from that of the species containing the gene for several reasons (Maddison 1997), including incomplete lineage sorting (ILS), duplication and loss (DupLoss for short), gene transfer, and hybridization. Species-tree inference is a central question in evolutionary biology and dealing with these sources of discordance is crucial. Many approaches have been proposed for species-tree inference, including gene trees–species tree coestimation (Liu 2008; Heled and Drummond 2010; An et al. 2013; Boussau et al. 2013; Szöllősi et al. 2015) and species-tree inference from sequence data (Bryant et al. 2012; De Maio et al. 2013; Chifman and Kubatko 2014). However, the most scalable approach has remained a two-step process: first infer gene trees independently from sequence data and then combine them using summary methods. The goal of a summary method is to find the species tree best explaining the gene trees according to a model of gene tree discordance. Although the ultimate goal is to develop summary methods modeling all sources of discordance, the literature has mostly focused on separate causes. 

A major family of summary methods focuses on duplication and loss processes producing multicopy gene trees (Hallett and Lagergren 2000; Ma et al. 2000; Wehe et al. 2008; Bansal et al. 2010; Chaudhary et al. 2010; Bayzid et al. 2013). Most of these summary methods rely on maximum parsimony reconciliation (Goodman et al. 1979) and aim at finding the species tree with the minimum reconciliation cost. Example methods include DupTree (Wehe et al. 2008), its later extension iGTP (Bansal et al. 2010; Chaudhary et al. 2010), DynaDup (Bayzid et al. 2013), and earlier similar dynamic programing algorithms (Hallett and Lagergren 2000). Other methods take a more agnostic approach and minimize the distance between species trees and the gene trees without necessarily invoking specific reasons for discordance. Example methods of this type include MulRF (Chaudhary et al. 2013) and *guenomu* (De Oliveira Martins et al. 2016). A recent result asserts that the optimal solution to the optimization problem solved by MulRF is indeed a statistically consistent estimate of the species tree under a generic duplication-only model of gene evolution (Molloy and Warnow 2019). These methods are mostly designed to handle duplication and loss, and although in simulations some have reasonable accuracy under ILS and gene transfer (Chaudhary et al. 2015), they have not been widely adopted.

Several summary methods target ILS as modeled by the multispecies coalescence (MSC) model (Pamilo and Nei 1988; Rannala and Yang 2003), and many of them are statistically consistent (e.g., Liu et al. 2009, 2010; Larget et al. 2010; Mossel and Roch 2010; Liu and Yu 2011; Wu 2012; Vachaspati and Warnow 2015; Sayyari and Mirarab 2016a). The most successful summary method for ILS has arguably been ASTRAL (Mirarab et al. 2014), which, due to its high accuracy (Giarla and Esselstyn 2015; Molloy and Warnow 2018; Ballesteros and Sharma 2019) and scalability (Mirarab and Warnow 2015; Yin et al. 2019), has been used to perform species-tree inference in numerous studies. ASTRAL, like several other methods (e.g., Larget et al. 2010; Chifman and Kubatko 2014; Sayyari and Mirarab 2016a), relies on dividing gene trees into unrooted four-taxon trees (called quartets), a feature that allows it to address ILS and may contribute to its high accuracy. ASTRAL, however, was designed to handle single-copy gene trees reconstructed from sets of orthologous genes. This limitation has restrained its application scope. As an example, two studies on plant transcriptomes had to discard thousands of available multicopy genes (Wickett et al. 2014; Leebens-Mack et al. 2019) and only use the 400–800 single-copy gene trees. A recent result by Legried et al. (2020) asserts that treating gene copies as alleles of a same gene, a feature ASTRAL supports (Rabiee et al. 2019), is a valid method under a parametric model of gene duplication and loss and *may* lead to accurate results. Du et al. (2019) have shown that random sampling of leaves works well empirically and Markin and Eulenstein (2020) have shown that method to be consistent under a model combining ILS and duplication and loss. Beyond ASTRAL, several methods have focused on dividing multicopy gene trees into single-copy genes without apparent duplications (e.g., Marcet-Houben and Gabaldón 2011; Scornavacca et al. 2011; Dunn et al. 2013; Yang and Smith 2014; Ballesteros and Hormiga 2016). However, to our knowledge, no quartet-based methods *designed* to handle duplication and loss currently exist. Extending quartet-based methods to multicopy gene trees while modeling orthology and paralogy is difficult.

We introduce the quartet-based species-tree inference method ASTRAL for PaRalogs and Orthologs (ASTRAL-Pro). Given a set of multicopy gene family trees, ASTRAL-Pro seeks to compute a single-copy tree (the species tree) maximizing the total similarity to the input gene trees. To define the similarity, we introduce a new measure of quartet similarity between single-copy and multicopy trees accounting for orthology and paralogy. Tests on an extensive set of simulated and real data sets provide evidence of ASTRAL-Pro’s robustness and accuracy.

## Results

We start by informally introducing the methodology underlying ASTRAL-Pro, leaving the formal definition and proofs to the Materials and Methods section. We will then compare the performances of ASTRAL-Pro with leading alternative methods on simulated and real data sets.

### ASTRAL-Pro Algorithm

#### Per-Locus Quartet Similarity

ASTRAL-Pro maximizes a measure of quartet similarity between a multicopy and a single-copy tree. Let us consider a rooted gene family tree where multiple leaves can have the same label (i.e., the species identifier). We need a principled way to compare this tree with a species tree where each species identifier appears once. The measure we define is based on several observations.

**Fig. 1.**
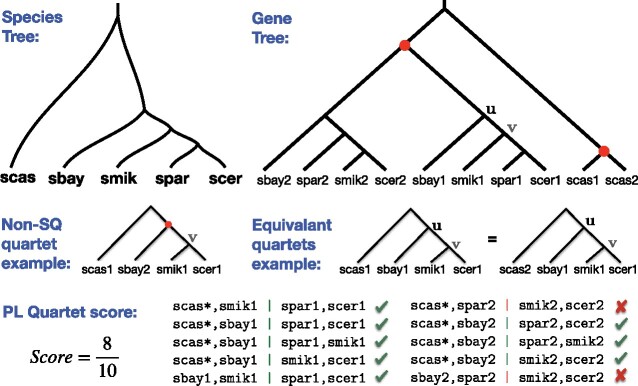
Per-locus quartet score. Example gene family tree from the fungi data set (Butler et al. 2009) restricted to five species and a potential species tree. Two nodes of the gene tree are tagged as duplication (red dots) and others as speciation. Quartet scas1, sbay1 | smik1, scer1 is anchored by nodes *u* and *v*, where *u* is the anchor LCA. Because the LCAs of any three leaves (*u* or *v*) are speciation nodes, this quartet is a SQ. Quartet scas1, sbay2 | smik1, scer1 is anchored by node *v* and a duplication (top red dot). Since the duplication node is the LCA of three leaves, this quartet is a non-SQ that does not count toward the per-Locus (PL) quartet score. Note *u* is the anchor LCA of both scas1, sbay1 | smik1, scer1 and scas2, sbay1 | smik1, scer1; thus, they form the equivalence class scas*, sbay1 | smik1, scer1. In this example, there are ten equivalence classes of SQ quartets, eight of which match the species tree; thus, the PL quartet similarity is 8. The goal of ASTRAL-Pro is to find the species tree that maximizes this score summed over all input trees.

- Internal nodes of the gene tree correspond to either duplication or speciation events; thus, we can *tag* nodes of the tree as speciation or duplication (Definition 1; see Materials and Methods). Although the true tagging is unknown, as we will see, it can be partially inferred (fig. 1).
- Each quartet of leaves in the gene tree defines two *anchor* nodes, and we refer to the Least Common Ancestor (LCA) of the two anchors as the *anchor LCA* (fig. 1). In a correctly tagged tree, a quartet has information about the speciation events only if it includes four distinct species and if the LCA of any three out of four leaves of the quartet is a speciation node (fig. 1). Thus, to define our measure of quartet similarity, we only include these speciation-driven quartets (SQs) and ignore the rest (Definition 2).
- All the SQs on the same four species that share the same anchor LCA must also share the same topology (Proposition 1). Thus, once we know the topology of one of them, the others do not provide new information. We call these SQs *equivalent* (Definition 4); in our quartet measure, we count them as one unit, and we consider them as part of the same quartet equivalence class. Moreover, we show that, for all equivalent quartets, the gene copies present at the current time all share the same ancestral locus at the time of the speciation event corresponding to the anchor LCA (Proposition 2) (see Materials and Methods for formal statements).

Based on these observations, we define the per-locus quartet score of a species tree *S* with respect to a gene family tree *G* with tagged internal nodes to be the number of quartet equivalence classes of *G* agreeing with *S* (Definition 5). We then define the Maximum per-Locus Quartet-score Species Tree (MLQST) for a set G of gene trees as the tree that has the maximum total per-locus quartet score with respect to G (Definition 6).

#### ASTRAL-Pro

As formalized in Theorem 1 in Materials and Methods, our new method is based on an efficient dynamic programing algorithm to find the MLQST tree. The ASTRAL-Pro algorithm, like ASTRAL, solves this problem restricted to a large search space *X*, defined heuristically using Algorithm 2. The running time of ASTRAL-Pro grows polynomially with the number of species, the number of genes, and the size of *X* (Claim 3). Finally, note that the per-locus quartet score is only defined for rooted and tagged gene trees. Since, in practice, gene trees are often unrooted and untagged, we also provide Algorithm 1 to tag and root gene trees using the parsimony principle.

#### Statistical Consistency and Local Support

In the presence of gene duplication and losses only, under the birth–death model called GDL proposed by Arvestad et al. (2009), Theorem 2 (Materials and Methods) states that ASTRAL-Pro is statistically consistent given correctly tagged and rooted error-free gene trees, even with partially correct rooting (see Claim 1). Under the MSC model and in the absence of gene duplication and gene loss, gene trees are single-copy. For single-copy gene trees, ASTRAL-Pro solves the same problem as ASTRAL and thus, like ASTRAL, it is a statistically consistent estimator of the species tree under the MSC model given a random sample of error-free gene trees (Mirarab et al. 2014). However, we do not currently have a proof of consistency of ASTRAL-Pro under models that combine GDL and ILS (see Discussions).

With correctly tagged error-free gene trees, differences in SQ topologies from the species tree must be due to processes other than GDL, such as ILS (Proposition 3). We use this observation to extend the local posterior probability (localPP) measure of branch support to multicopy gene trees (Definition 8).

### Accuracy of ASTRAL-Pro in Simulations

We first test ASTRAL-Pro (A-Pro for short) against two leading summary methods: MulRF (Chaudhary et al. 2013) (optimizing an extension of the RF distance [Robinson and Foulds 1981] to multilabeled trees) and DupTree (Wehe et al. 2008) (minimizing the duplication reconciliation cost [Maddison 1997]). We also compare A-Pro with ASTRAL-multi (Rabiee et al. 2019), which is the feature of ASTRAL designed for handling multiple alleles (as opposed to multiple copies); although ASTRAL-multi is not designed for multicopy data, we include it because of recent theoretical results showing that it is consistent under the GDL model (Legried et al. 2020). We compare the methods in terms of the accuracy of the species tree topology that they produce.

In our tests, we use two simulated data sets, one called S25, which is new to this study, and one called S100 from Molloy and Warnow (2019), which is based on a real fungal data set (Butler et al. 2009; Rasmussen and Kellis 2012). Both data sets were created by 1) simulating true gene trees under the DLCoal model, which is a unified model of ILS and gene duplication and loss (Rasmussen and Kellis 2012), 2) simulating a sequence alignment from each true gene family trees, and 3) estimating a gene tree from each gene alignment. In S25, we varied parameters that control the rate of duplication ($\lambda_{+}$), the rate of loss ($\lambda_{-}$), the ILS level, the number of species (*n*), and the number of genes (*k*) (table 1). We also varied alignment length, which effectively varied the level of gene tree estimation error. The S100 data set also varies all these parameters, except *n*. Thus, we simulate effects of ILS, duplication and loss, and gene tree estimation error (see Materials and Methods for details).

**Table 1.** Simulation Settings for S25 Data Set with Varying Parameters.

Condition	Parameter Ranges
Default model	*n* = 25; *k* = 1,000; τ∼LN(21.25;0.2)
	$\lambda_{+}=4.9\times 10^{-10}$; $\lambda_{-}=\lambda_{+}$; $N_{e}=4.7\times 10^{8}$
	C≈5; ILS ≈ 70%
	MGTE = 15% (500 bp) or 36% (100 bp)
Varying $\lambda_{+},\lambda_{-}$ (DupLoss rate)	$\lambda_{+}\in \{4.9,2.7,1.9,0.52,0\}\times 10^{-10}$
	$\lambda_{-}\in \{1,0.5,0.1,0\}\times \lambda_{+}$; C≈{5,2,1,0.2,0}
Varying $\lambda_{+},N_{e}$ (dup rate, ILS)	$\lambda_{+}\in \{4.9,1.9,0\}\times 10^{-10}$;
	$N_{e}\in \{4.7,1.9,0.48,0.0001\}\times 10^{8}$
	ILS ≈{70,52,20,0}%; C≈{5,1,0}
	MGTE ≈{15,15,15,16}% (500 bp) or
	{36,36,36,35}% (100 bp) as *N*_e_ changes
Varying *n*	n∈{10,25,100,250,500}
	MGTE ≈{15,15,17,18,18}% (500 bp)
	or {34,36,40,43,43}% (100 bp)
Varying *k*	k∈{25,100,250,1,000,2,500,10,000}

Note.—See supplementary table S1, Supplementary Material online, for full parameters and supplementary figures S1–S6, Supplementary Material online, for full statistics. *n*, number of ingroup species; *k*, number of genes; *τ*, tree height in generations; $\lambda_{+}$, duplication rate; $\lambda_{-}$, loss rate; *N*_e_, haploid effective population size. We estimated the following empirically. *C*, mean number of copies per species minus one when $\lambda_{-}=0$ and *n *=* *25; ILS, mean RF distance between true gene trees and the species tree when $\lambda_{+}=0$; MGTE, mean RF distance between true and estimated gene tree when $\lambda_{+}=0$.

#### S25 Data Set

##### Controlling Duplication and Loss Rates

**Fig. 2.**
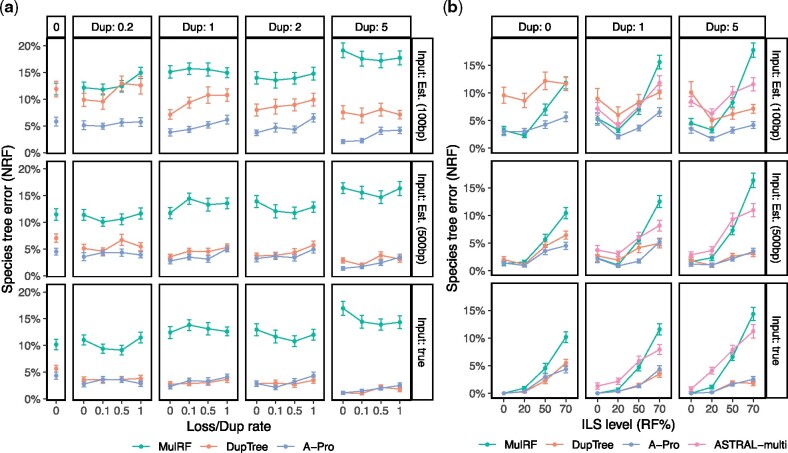
Species tree error on the S25 data set for *n *=* *25 ingroup species, *k *=* *1,000 gene trees, and both true and estimated gene trees from 100 and 500 bp alignments. (*a*) Controlling duplication rate (box columns; labeled by *C*) and the loss rate (*x*-axis; ratio of the loss rate to duplication rate). (*b*) Controlling the duplication rate (columns; labeled by *C*) and the ILS level (*x*-axis; NRF between true gene trees and the species tree for $\lambda_{+}=0$). A-Pro and ASTRAL-multi are identical with $\lambda_{+}=0$. See table 1 for parameters and supplementary figure S7, Supplementary Material online, for iGTP-DupLoss.

We begin by describing the results of experiments that vary the duplication and loss rates ($\lambda_{+},\lambda_{-}$) (fig. 2*a*). On true gene trees, A-Pro and DupTree are essentially tied in terms of accuracy, except for the case with no duplication and loss where A-Pro is slightly more accurate. Overall, the accuracy of A-Pro and DupTree is statistically indistinguishable under these conditions (*p* value = 0.79 according to a multivariate analysis of variance (ANOVA) test). Increasing $\lambda_{+}$*reduces* error ($p<10^{-5}$), perhaps because additional copies provide more information, akin to increasing the number of loci. Despite statistically significant increases (*p *=* *0.006) in error as $\lambda_{-}$ increases, both methods are quite robust to loss rates, losing at most 1.5% accuracy on average when $\lambda_{-}=\lambda_{+}$ compared with no losses. MulRF has much higher error than other two methods, with errors that range between 10% and 17% across model conditions (we remind the reader that all these conditions exhibit high ILS, a process that MulRF ignores).

On estimated gene trees, the pattern changes, and the error of DupTree increases dramatically, whereas A-Pro remains relatively accurate. When $\lambda_{+}=\lambda_{-}=0$, DupTree has on average an 11.5% error, whereas A-Pro has only a 4.5% error for 500 bp. Adding duplications helps both methods, but A-Pro remains more accurate. For example, with 100-bp input gene trees (i.e., high estimation error), DupTree has errors between 50% and 260% higher than A-Pro. With 500-bp input (i.e., low-error gene trees), differences are statistically significant ($p<10^{-5}$) but are more modest in magnitude (across conditions, DupTree has a median of 28% higher error). The relative accuracy of A-Pro and DupTree is not a function of $\lambda_{-}$ (*p *=* *0.8) but may depend on $\lambda_{+}$ (*p *=* *0.05).

In terms of running time, on the default model condition, we observe that A-Pro is the fastest method, taking less than a minute on this data set, followed closely by DupTree (supplementary fig. S8, Supplementary Material online).

##### Controlling the Level of ILS

As we change the ILS level (table 1), the reason for the poor performance of MulRF becomes clear (fig. 2*b*). Without ILS, MulRF has excellent accuracy, often matching A-Pro and beating DupTree on low-error gene trees. As the ILS level increases (especially above 20%), the accuracy of MulRF deteriorates quickly. Overall, ILS has the strongest effect on accuracy (*p* $\ll 10^{-5}$) but its impact on methods varies ($p\ll 10^{-5}$). DupTree seems as tolerant of ILS as A-Pro, despite the fact that DupTree is not designed specifically for ILS, and both methods are much more tolerant of ILS than MulRF. Nevertheless, once again, DupTree shows extreme sensitivity to gene tree error.

To summarize, DupTree is relatively tolerant of ILS but less tolerant of gene tree error; MulRF is tolerant of gene tree error but not of ILS; A-Pro is quite robust to both.

##### Controlling the Number of Genes and Species

**Fig. 3.**
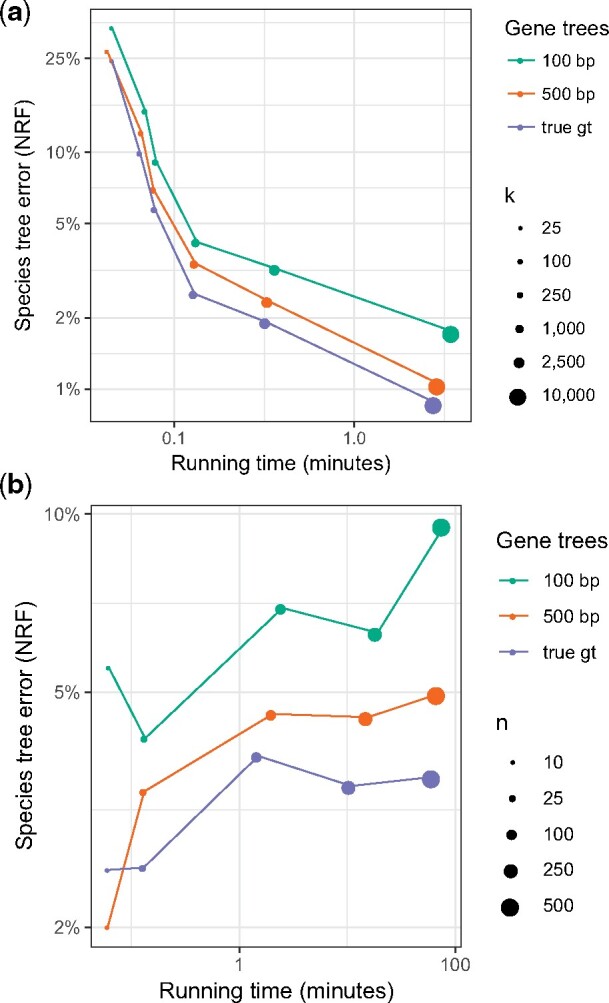
Accuracy (*y*-axis) and running time (*x*-axis) of A-Pro as the number of genes *k* (*a*) or the number of species *n* (*b*) changes. Both axes are in log-scale. As *k* increases, accuracy increases (see also supplementary figure S9, Supplementary Material online).

Increasing the number of genes *k* in the most difficult model condition (i.e., high $\lambda_{+},\;\lambda_{-}$, and ILS) results in continued improvement in accuracy for A-Pro for every value we tested up to $k=10^{4}$ (fig. 3*a*). With true gene trees, the error reduces from 26% with *k *=* *25 to below 1% with $k=10^{4}$. Even with less accurate gene trees, the error reduces to below 2% with increased numbers of genes. Increasing *k* increases running time, which empirically grows proportionally with $k^{1.4}$ (supplementary fig. S9*a*, Supplementary Material online). Nevertheless, using 28 cores, the running time was never more than 3.5 min even with $k=10^{4}$.

Increasing *n* from 25 to 500 shows that A-Pro is relatively robust to a large number of species (fig. 3*b*). With true gene trees, the error ranges between 2.5% with 10 species and 3.5% with 500 species. With estimated gene trees, error ranges between 4.1% and 9.5% (for 100 bp) and between 2% and 5% (for 500 bp). Note that as *n* increases, the gene tree error also increases (table 1 and supplementary fig. S6, Supplementary Material online). The running time of A-Pro increases roughly quadratically with *n* (supplementary fig. S9*b*, Supplementary Material online) but is below 2 h (given 28 cores) even for *n *=* *500 (*k *=* *1,000).

#### S100 Data Set

**Fig. 4.**
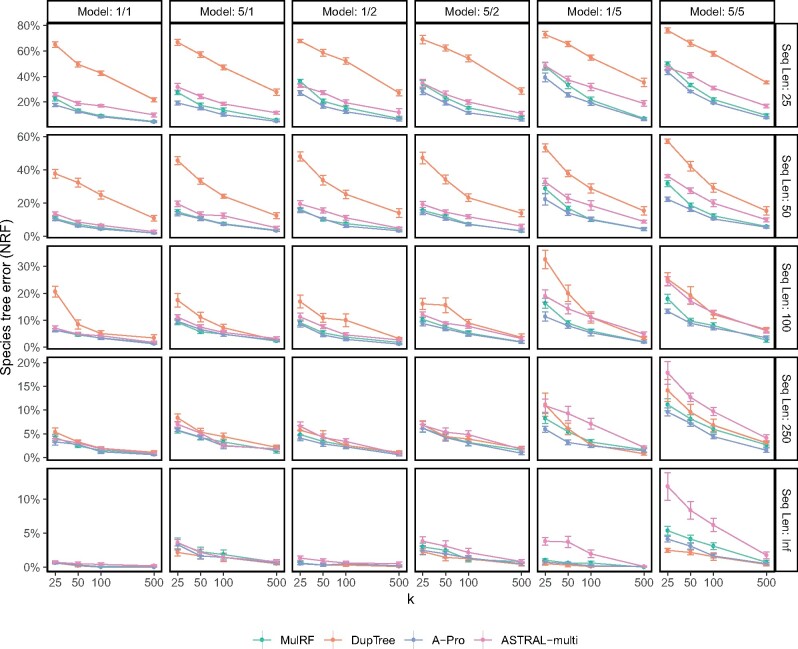
Species tree error on S100 data set. We compare the species tree error of the four methods, showing mean and standard error over ten replicates for each model condition, with varying numbers of genes (*k*) and sequence lengths (with Inf signifying true gene trees). Model conditions are labeled as *a*/*b* where *a* is the level of ILS (1 or 5) and *b* is the duplication/loss rate (1, 2, or 5).

Patterns of performance on the S100 data set are consistent with the S25 data set (fig. 4). DupTree is highly accurate with true gene trees and gene trees with low estimation error but quickly degrades in accuracy as gene tree error increases. MulRF is less sensitive to gene tree error but is more sensitive to the ILS level (which is always moderate or low on this data set). As in S25, here, we see that using ASTRAL-multi to handle duplication and loss is not beneficial.

A-Pro works the best overall, ranking first in terms of mean error (rounded to two significant digits) in 105 out of 120 test conditions and ranking second in 14 of the 15 remaining cases (supplementary table S2, Supplementary Material online). Many of the conditions where A-Pro is ranked second are among those with true gene trees where DupTree works great. The second best method overall is MulRF, which is not surprising given the low ILS levels in this data set. As expected, all methods are helped with increased numbers of genes; however, even with 500 genes, differences in accuracy remain, especially with shorter gene sequences.

### Accuracy on Biological Data Sets

#### Plant (1KP) Data Set

We reanalyze the transcriptome data set of 103 plant species, which was previously analyzed by Wickett et al. (2014) using 424 single-copy gene trees using ASTRAL. The original study had also inferred 9,683 multicopy gene trees with up to 2,395 leaves for 80 of the 103 species and three other genomes (a total of 83). However, due to a lack of suitable species tree methods, these gene trees were left unused (Materials and Methods). Here, we analyze all 9,683 multicopy gene trees using A-Pro.

**Fig. 5.**
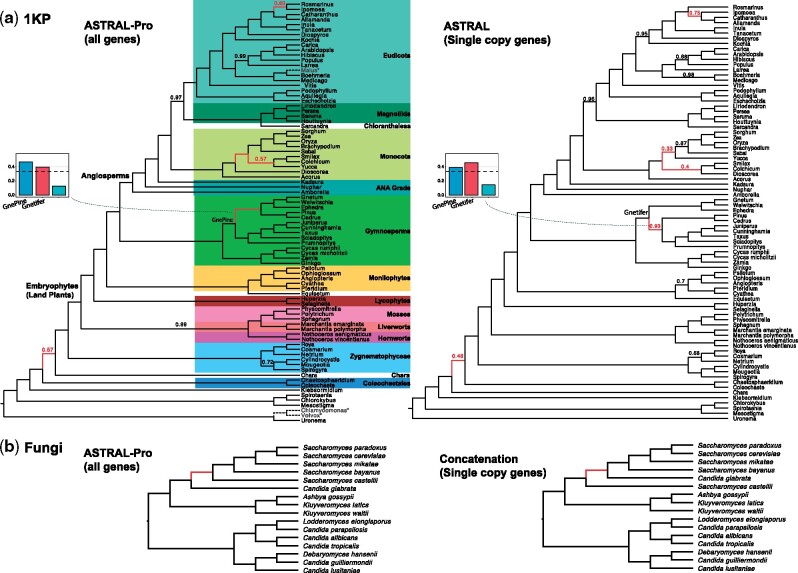
Biological data set. (*a*) Plant data set (1kp). Right: ASTRAL on 424 single-copy gene trees. Left: ASTRAL-Pro on 9,683 multicopy gene trees. Three genomes (noted by * and dashed lines) were present in multicopy data set but not in the single-copy data. The single-copy tree includes 23 species that were not in the multicopy data and are pruned from the species tree (localPP support is recomputed using gene trees pruned to the 80 common species). Five branches (red) differ between the two trees. LocalPP support shown except when equal to 1. For the main highly supported conflict (Gnetifer vs. Gnepine), we show quartet support of alternative topologies among single-copy gene trees using DiscoVista (Sayyari et al. 2018). (*b*) Fungi data set. Right: Concatenation of 706 single-copy gene trees with the red branch enforced as a constraint (Butler et al. 2009). Left: ASTRAL-Pro on 7,280 multicopy gene trees.

A-Pro on multicopy gene trees returns a species tree (fig. 5*a*) similar to the single-copy ASTRAL tree reported by the original study but with five differences. In contrast, DupTree differs from the ASTRAL tree in 33 out of 77 branches (21/77 for iGTP-DupLoss) and violates many known biological relationships (supplementary fig. S10, Supplementary Material online). A-Pro has higher localPP than ASTRAL (e.g., four vs. eight branches with localPP below 0.95). The A-Pro tree is consistent with ASTRAL for major groups, including placing *Zygnematales* (not *Chara*) as sister to all land plants, the placement of *Amborella* as sister to the rest of angiosperms, and the monophyly of Bryophytes (liverworts, mosses, and hornworts). Some of these consistencies with ASTRAL (e.g., monophyly of Bryophytes) are in contrast to the concatenation analyses of single-copy genes, as reported by Wickett et al. (2014).

Changes between the ASTRAL and A-Pro trees mostly have low support. In A-Pro, unlike ASTRAL, Rosmarinus and Ipomoea are grouped together (albeit, with 0.6 localPP support), which is likely the correct result as these species are in the same order (Lamiales). The ASTRAL tree has only 0.75 localPP for dividing this order. The position of genus Yucca has low support in the ASTRAL tree and has changed in the A-Pro tree. Interestingly, a recent update to this transcriptome analysis using 1,124 species (Leebens-Mack et al. 2019) (which samples close genera *Asparagales* and *Liliales*) finds Yucca in a position identical to A-Pro. Another change is the relative position of *Coleochaetale* and *Chara* which has low localPP in both trees. Most consequentially, the main highly supported change is that A-Pro, unlike ASTRAL, recovers the GnePine hypothesis (i.e., combining Gnetales and Pinaceae) with 1.0 localPP. This hypothesis is supported by several studies (Burleigh and Mathews 2004; Zhong et al. 2010, 2011; Laurin-Lemay et al. 2012) and all concatenation analyses from Wickett et al. (2014). Examining quartet frequencies for single-copy gene trees around this branch, we see that the second and third most frequent quartets do not match (fig. 5*a*) and are skewed toward GnePine; this pattern is not consistent with ILS as the main source of discordance, and may suggest other processes such as hybridization. However, multicopy gene trees also show a similar pattern, with support for GnePine and Gnetifer swapped.

#### Fungal Data Set

We reanalyze a data set of 16 yeast species with 7,280 multicopy gene families available from Butler et al. (2009). To obtain the species tree, the original study used only 706 one-to-one orthologs with concatenation and did not use multicopy gene trees in species tree inference (Materials and Methods). We used all amino acid multicopy gene families as input to A-Pro.

The A-Pro species tree has 1.0 localPP everywhere and matches the published species tree except for one branch (fig. 5*b*). The position of *Saccharomyces castellii* as sister to *Candida glabrata* and the *Saccharomyces* group in the original study was enforced by a constraint in the ML search because the unconstrained analyses did not recover the relationship the authors expected. This enforced constraint was justified based on genome rearrangement and syntenic conservation, but was not recovered in the concatenation analyses. In the A-Pro tree, *Candida glabrata* is at the base of this clade, matching the unconstrained concatenation analysis. Salichos and Rokas (2013) also recovered the same topology as A-Pro and used this branch as an example of relationships that challenge phylogenomics. Although gene synteny evidence suggests that A-Pro may be finding the wrong resolution, it is worth highlighting that it matches trees inferred using substitution models.

## Discussions

We introduced A-Pro, a summary method for combining multicopy gene trees. By allowing the use of multicopy gene trees, A-Pro enables a manyfold increase in the number of genes used in phylogenomic analyses. Note that neither concatenation nor ASTRAL (the dominant methods used by practitioners) is able to use multicopy genes. The main set of methods available for multicopy analyses are the coestimation methods (e.g., Szöllosi et al. 2012; Boussau et al. 2013; Szöllősi et al. 2013). However, these methods, although accurate, are inherently less scalable than summary methods. A-Pro provides a scalable yet accurate alternative to these coestimation methods.

**Fig. 6.**
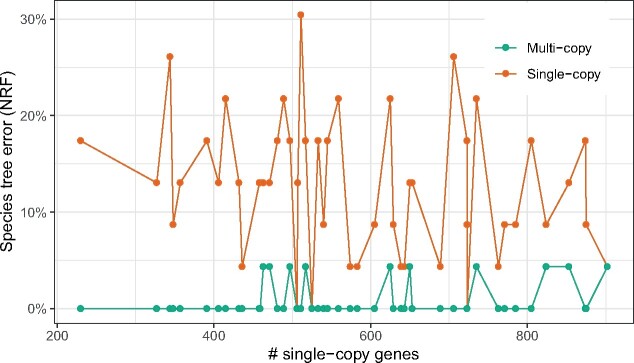
Accuracy of the estimated species tree (*y*-axis) versus the number of single-copy genes (*x*-axis) across all 50 replicates of the S25 data set with *k *=* *10,000 gene trees (from the experiment varying *k*). The “Multicopy” line, representing A-Pro, is using all gene trees, whereas the “Single-copy” line, representing ASTRAL, is only using the single-copy gene trees.

As an example for testing the advantage of using all multicopy gene trees, we revisit the simulated S25 data set with $k=10^{4}$ multicopy gene trees. Among the 10^4^ gene trees, we have between 200 and 900 single-copy gene trees across our 50 replicates (the variation is due to stochastic differences). An alternative to using ASTRAL-Pro is to use normal ASTRAL on single-copy gene trees. Comparing ASTRAL on single-copy gene trees and ASTRAL-Pro on all 10^4^ multicopy gene trees shows a great loss of accuracy as a result of the filtering (fig. 6). Our simple filtering strategy, keeping all single-copy gene trees, does not consider orthology but is not dramatically different from the approach used by many (e.g., Leebens-Mack et al. 2019; Wickett et al. 2014). Despite the potential for paralogy in single-copy genes, the example shows the negative impact of gene filtering. This observation is consistent with prior results that have established a close link between the accuracy of summary methods and the number of input genes both in practice (for an overview, see Mirarab 2019) and in theory (Shekhar et al. 2018).

A-Pro is based on a per-locus quartet-based measure of similarity between multicopy gene trees and a species tree. The measure relies on internal nodes of gene trees being tagged as speciation or duplication. Somewhat counterintuitively, despite being a quartet measure, it needs *partially* rooted trees (Claim 1). The measure defines an equivalence relationship on quartets and counts each equivalence class only once, avoiding double-counting quartets that are bound to have identical topologies. Avoiding double-counting is at the heart of the approach and likely is a main reason behind its high accuracy on the simulated and empirical data we tested.

Quartet-based methods for handling multicopy gene trees are not abundant. Besides our method, one can attempt to sample single-copy gene trees, an approach that shows promise but fails to model orthology/paralogy (Du et al. 2019). Legried et al. (2020) recently provided theoretical and empirical evidence that simply treating gene copies as alleles may be sufficient. We showed that this alternative, although attractive in theory, is less accurate and less scalable than A-Pro. We are unaware of other quartet-based species-tree inference methods for multicopy input. Nevertheless, our approach is not the only one that can be imagined and future work should explore other quartet metrics.

To get rooted and tagged gene trees, we used the maximum parsimony principle, with duplication and loss each penalized equally and deep coalescence not penalized at all (methods). The algorithm we use is not guaranteed to find the correct tags or the root under complex scenarios involving gene duplication and subsequent losses. Thus, the consistency results under the GDL model should be interpreted with this caveat in mind. A-Pro may be statistically consistent even when gene trees are imperfectly rooted and tagged, but we leave this to be determined in future work. Furthermore, there is a large literature on various ways of tagging and rooting gene trees (e.g., Durand et al. 2006; Bansal et al. 2013; Jacox et al. 2016), including other penalties for the duplication and loss events (e.g., there is a suggestion of losses having half the penalty of duplications [David and Alm 2011]). It may also be possible to improve tagging of gene trees using probabilistic orthology inference (Arvestad et al. 2004; Sennblad and Lagergren 2009) or using synteny information (Bourque et al. 2005; Chauve et al. 2013). However, these methods often require a species tree. It may be possible to use A-pro in an iterative fashion, where the species tree is inferred, gene trees are retagged and rerooted, and a new species tree is inferred. Future work should explore these approaches.

A-Pro, like other summary methods, depends on accurate input trees. Although A-Pro is more robust to gene tree error than alternatives, combining it with coestimation (Boussau et al. 2013) or gene tree correction (Lafond et al. 2013, 2014; Wu et al. 2013; Scornavacca et al. 2015; Noutahi et al. 2016; El-Mabrouk and Noutahi 2019) may further improve its accuracy. Future work should also explore extending A-Pro to multifurcating input gene trees because contracting low support branches may help dealing with gene tree error (Zhang et al. 2018).

ASTRAL-Pro, which maximizes the per-locus quartet score, is statistically consistent under the MSC model (when given single-copy gene trees as input) and under a GDL model (when given multicopy gene trees as input). This makes one hope that it may also be consistent under both causes of discordance combined. The DLCoal model (Rasmussen and Kellis 2012) accounts for ILS, duplication, and loss. Under this model, each duplication immediately creates a daughter locus, which is unlinked from the parent locus; the duplication event gets fixed in all species. Gene trees are seen as generated by first producing a locus tree via a birth–death process that runs on the species tree and then running an MSC process on the locus tree. Because the loci are considered as unlinked, the coalescence processes occur independently between the parent and daughter loci (but the daughter MSC process is “bounded” at the time of duplication). Interestingly, a new paper has suggested that simply selecting one copy of each gene at random and feeding the resulting gene trees to ASTRAL would be consistent under the DLCoal model (Markin and Eulenstein 2020). Due to the independence of loci, dividing a multicopy gene family into its constituent loci can give us distributions on gene tree topologies that behave similarly (though not identically) to the MSC model. The per-locus metric *seeks* to count quartet topologies across loci as they existed at the time of speciation events relevant to a quartet (i.e., at the time of the anchor LCA). When successful, it counts only topologies that are drawn from independent coalescent processes. However, complicated scenarios involving a combination of duplications, losses and ILS can lead to incorrectly tagged gene trees. These scenarios create complications for theoretical proofs. Although our simulations were performed under the DLCoal model, we leave it to the future to study whether ASTRAL-Pro is statistically consistent under the DLCoal model.

Our simulations, which all followed the DLCoal model, do not consider some relevant biological scenarios. Examples include whole-genome duplication events, interlocus gene conversion, and hemiplasy of duplication and loss events (Li et al. 2020). Since ASTRAL-Pro is nonparametric (i.e., does not assume rates of duplication), we predict that whole-genome duplication events do not impose a major obstacle. The impact of interlocus gene conversion is much harder to predict and needs careful testing. Future work should study ASTRAL-Pro under these more complex scenarios of duplication and loss.

## Materials and Methods

### The Algorithm

Proofs of all propositions, lemmas, and claims can be found in supplementary Proofs, Supplementary Material online.

#### Notations and Definitions

Let S be a set of *n* species. Let us suppose that we are given a set of binary gene trees G, and, for each tree G∈G with leaf set ${ℒ}_{G}=\{1\ldots m_{G}\}$, we have a mapping $\alpha_{G}:{ℒ}_{G}\to S$ specifying in which species each gene is sampled. For a rooted tree *G*, we denote the set of internal nodes in *G* by *I*(*G*), and, for each u∈I(G), we define ${ℒ}_{G}(u)$ as the set of leaves below *u*. We define two short-hands: $\alpha_{G}(A)=\{\alpha_{G}(i):i\in A\}$ for $A\subset {ℒ}_{G}$ and $\alpha_{G}(u)=\alpha_{G}({ℒ}_{G}(u))$ for a node *u* (i.e., all species labels corresponding to a set *A* of gene tree leaves and all species labels under a gene tree node *u*, respectively). The notation G↾A denotes *G* restricted to the set *A*.

We let Ω(G) be the multilabeled tree obtained by replacing each leaf $l\in {ℒ}_{G}$ with $\alpha_{G}(l)$. Multiple copies of the same species in a gene tree *G* may be created by gene duplication. Note that we ignore other processes such as transfers, gene conversion, and hybridizations. We assume that each duplication creates a new genomic locus (i.e., a position along the genome) and therefore, each locus, except the original one, has a parent locus (which may or may not have survived to the present day). Thus, each element of ${ℒ}_{G}$ can be theoretically mapped to its parent locus, allowing us to “trace” the locus of each leaf to its ancestors.

**Fig. 7.**
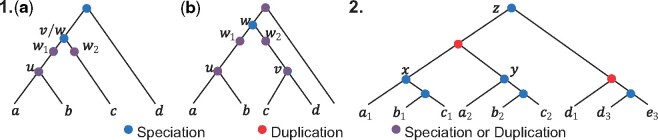
(1) An example of a quartet Q={a,b,c,d} with (*a*) unbalanced topology (Q ∠ G) and (*b*) balanced topology (Q⊥G). Anchors are *u* and *v*, and *w* is the anchor LCA. Although *w* has to be a speciation for *Q* to be considered a SQ, *u* and *v* (when different from *w*) can be either speciation or duplication. (2) An example of equivalence classes. Three equivalence classes are anchored on *z*: all eight quartets of the form $\left\{a_{i},b_{j},d_{k},e_{3}\right\}$, of the form $\left\{a_{i},c_{j},d_{k},e_{3}\right\}$, and of the form $\left\{b_{i},c_{j},d_{k},e_{3}\right\}$, all with balanced topology. Anchored on *x*: two equivalence classes with unbalanced topology: $\left\{a_{1},b_{1},c_{1},d_{1}\}∼\{a_{1},b_{1},c_{1},d_{3}\right\}$ and $\left\{a_{1},b_{1},c_{1},e_{3}\right\}$. Anchored on *y*: two equivalence classes: $\left\{a_{2},b_{2},c_{2},d_{1}\}∼\{a_{2},b_{2},c_{2},d_{3}\right\}$ and $\left\{a_{2},b_{2},c_{2},e_{3}\right\}$.

In each gene tree *G*, we refer to a subset *Q* of four distinct elements of ${ℒ}_{G}$ as a quartet. The subtree of a fully resolved tree *G* restricted to a quartet *Q* exhibits two degree-three nodes. We refer to these nodes as *anchors of Q on G*. As shown in figure 7, for a rooted tree *G* and for a quartet *Q*, up to label permutations, G↾Q can only have two topologies: an *unbalanced* one (when one anchor descends from the other), denoted as Q ∠ G, and a *balanced* one (otherwise), denoted as Q⊥G. We say a tripartition $\left(P_{1},P_{2},P_{3}\right)$ of S “can anchor” a quartet *Q* of *G* iff $\forall_{i}:P_{i}\cap \alpha_{G}(Q)\neq \emptyset$.

Definition 1 (Tagged trees). We say that a rooted tree *G* is tagged if every internal node is tagged either as duplication or as speciation. A node *u* with children *u*_1_ and *u*_2_ can be tagged as speciation only if the sets $\alpha_{G}(u_{1})\;\text{and}\alpha_{G}(u_{2})$ are mutually exclusive.

We note that these labels may or may not correspond to real speciation and duplication events. In particular, when loci coalesce before duplication events, a correct tagging corresponding to actual events may not be possible.

#### Per-Locus Quartet Score

Definition 2 (SQ). A quartet *Q* on a rooted tagged gene tree *G* is called a SQ iff $|\alpha_{G}(Q)|=4$ and the LCA of any three out of four leaves of *Q* is a speciation node. Equivalently, a quartet with topology ab|cd is a SQ if and only if its genes are all contained in different species and the LCA of either *a* or *b* with either *c* or *d* is tagged as speciation. Let Σ_*G*_ denote the set of SQs in *G*.

Definition 3 (Quartet anchor LCA). Let *u* and *v* be anchors of a quartet *Q* on a *rooted* tree *G*. We refer to the LCA of *u* and *v* as the *anchor LCA* of *Q* on *G* and denote it as $\psi_{G}(Q)$.

The last definition is central to our approach. Note that anchors of a SQ can be speciations or duplications (fig. 7) and thus SQs are not simply quartets with anchors being speciation nodes. Instead, they are quartets with a topology predetermined by the speciation event represented by the anchor LCA, regardless of subsequent duplications and losses. Such subsequent duplications and losses may lead to multiple quartets being associated with the same speciation event. Since these events include no new information on the speciation event, we count only SQs toward the quartet score of a species tree and weight them in a nontrivial way to avoid double-counting.

Definition 4 (Equivalent SQs). Two SQs on the same four species are *equivalent* if they have the same anchor LCA; that is, for two SQs, $Q_{1}∼Q_{2}\Leftrightarrow \alpha_{G}(Q_{1})=\alpha_{G}(Q_{2})\wedge \psi_{G}(Q_{1})=\psi_{G}(Q_{2})$.

Proposition 1. *If* $Q_{1}$*and* $Q_{2}$*are equivalent SQs on G, then* $\Omega (G↾Q_{1})$*and* $\Omega (G↾Q_{2})$*are isomorphic.*

Thus, equivalent SQs have the same quartet topology when mapped to species. Proposition 1 tells us that equivalent SQs do not provide any extra information on the speciation event, and therefore, it is reasonable to count all equivalent SQs as one unit when computing the quartet score of a species tree. This intuition is backed by the following proposition:

Proposition 2. *Assuming a correctly rooted tagged tree G, for all equivalent SQs with a shared anchor LCA w, the three (in the unbalanced case) or four (in the balanced case) quartet leaves below w will all share an ancestral locus at the time of the speciation event corresponding to w.*

We can now provide a natural definition of the quartet score. The equivalence relation (Definition 4) partitions all quartets in equivalence classes and, by Proposition 1, for each equivalence class, we can define a unique quartet tree labeled by S. By Proposition 2, each class corresponds to an ancestral locus. We can denote each equivalence class in *G* as a pair, consisting of the set of species and the anchor node $\left(\alpha_{G}(Q),\psi_{G}(Q)\right)$.

Definition 5 (Per-locus Quartet Score). The per-locus quartet score of a species tree *S* with respect to a rooted tagged gene tree *G* is the number of equivalent quartet classes that match the *S* topology. More formally, q(S,G) is defined as:

$$|\{(\alpha_{G}(Q),\psi_{G}(Q)):Q\in \Sigma_{G},\Omega (G↾Q)≃S↾\alpha_{G}(Q)\}|\;.$$

The PL quartet score of *S* with respect to a set of tagged gene trees G is $q(S,G)=\underset{G\in G}{\Sigma }q(S,G)\;.$

Note that this definition gracefully handles missing data; gene family trees that do not include a specific species will not contribute quartets that include that species.

Definition 6 (MLQST problem). Given a set of rooted tagged gene trees G, find the species tree that maximizes the PL quartet score with respected to input gene trees, that is, $\text{arg}{\text{max}}_{S}q(S,G)$.

Finally, note that although the PL quartet score depends on rooting and tagging, it is robust to *some* changes in the root placement; thus, the tree needs to be only partially rooted.

Claim 1. *If all nodes on the path between the root r and a node u are tagged as speciations, changing the root to any branch on the path does not alter the PL quartet score.*

### Solving the MLQST Problem

We start by briefly describing the ASTRAL algorithm to solve a related problem (the Maximum Quartet Support Species Tree [MQSST] problem), and then describe how we extend this approach to the MLQST problem.

#### Background: ASTRAL on Single-Copy Gene Trees

Note that, a node in a binary single-copy unrooted species tree forms a tripartition of S that implies the topology for all quartets anchored at that node, and this observation is at the base of the scoring scheme of ASTRAL. More formally, let $P=P_{1}|P_{2}|P_{3}$ and $M=M_{1}|M_{2}|M_{3}$ be two tripartitions, and let $I_{ij}=|M_{i}\cap P_{j}|$. Any species tree that displays *P* will share a certain number of quartets with any gene tree that displays *M*, and we call this number *QI*(*P*, *M*) (calculations below extends to multifurcations if *M* is a *d*-partition). Defining *B*_3_ as the set of all permutations of {1, 2, 3}, Mirarab et al. (2014) showed:

(1) $$\begin{array}{c} W(P)=\frac{1}{2}\sum_{G\in G}\sum_{M\in P(G)}QI(P,M)\;\;\;\;\text{where}\;\;\;\;\\ QI(P,M)=\frac{1}{2}\sum_{(i,j,k)\in B_{3}}I_{i1}I_{j2}I_{k3}(I_{i1}+I_{j2}+I_{k3}-3) \end{array}$$

and P(G) is the set of partitions representing internal nodes of *G*. The quartet score of a species tree is simply the sum of the weights of its tripartitions. The division by half in *W*(*P*) is necessary because the sum counts each shared quartet twice (once at each anchor).

ASTRAL finds the tree *S* that maximizes the quartet score using dynamic programing. It recursively divides S into subsets, in each step, choosing the division that maximizes the sum of the weights. To avoid exponential running time, instead of considering all ways of partitioning a set A⊂S into A′ and A∖A′, it constrains the search space to a given set of bipartitions. Let *X* be this set and X′={A:A|(S∖A)∈X} and Y={(C,D):C∈X′,D∈X′,C∩D=∅,C∪D∈X′}. The quartet score of an optimal subtree on the cluster *A*, denoted as *V*(*A*), is

(2) $$V(A)={\underset{(A\prime ,A\setminus A\prime )\in Y}{\max}}_{}V(A\prime )+V(A\setminus A\prime )+W(A\prime |(A\setminus A\prime )|(S\setminus A)),$$

where V({a})=0 for all leaves a∈S. This value can be computed recursively, and the optimal tree for V(S) is the ASTRAL tree.

#### ASTRAL-Pro Algorithm

We extend here ASTRAL to multicopy gene trees. The input to the new method, called ASTRAL-Pro, is a set of rooted tagged gene trees. This extension involves three changes in the way the weight *w* is computed: 1) To handle multicopy gene trees, when computing the tripartition associated with each node, we use *α_G_* to map labels to S. Note that, in a tripartition $M=M_{1}|M_{2}|M_{3}$, the *M_i_* are *sets* and not *multisets* so multiple copies of the same species are considered only once. 2) We change the weight calculation *W*(*P*) so that each equivalence class of quartets is counted once instead of twice (only at its LCA anchor). 3) When computing *w*, we only sum over internal nodes tagged as speciations. In addition, two changes to the algorithm procedure are needed: we need to root and tag gene trees and properly define the set *X* for multicopy trees. We now detail these changes.

##### Weight Calculation

Let *G* be a rooted tagged gene tree, *w* an internal node of *G* tagged as speciation and $P=(P_{1}|P_{2}|P_{3})$ a tripartition of S.

Definition 7. For a species tree tripartition *P* and a SQ equivalence class that has the LCA anchor *w* in a gene tree *G*, we say that the SQ *is mapped from left to P* iff for each quartet *Q* in the equivalence class 1) *P* can anchor *Q* and 2) the leaves *a* and *b* under the anchor of *Q* that appear first in a postorder traversal of *G* (e.g., *u* in fig. 7) both map to the same side of *P* (i.e., $\alpha_{G}(a)\in P_{i},\alpha_{G}(b)\in P_{i}$ for some 1≤i≤3).

We denote such quartets by $Q\overset{w}{\to }P$.

We now state a set of lemmas, followed by the main result.

Lemma 1. *If* $Q_{1}∼Q_{2}$*and* $Q_{1}\overset{w}{\to }P$*, then* $Q_{2}\overset{w}{\to }P$.

Lemma 2. *For a speciation node w with left child w_1_ and right child w_2_, let* $M_{1}=\alpha_{G}(w_{1}),\;M_{2}=\alpha_{G}(w_{2})$*and* $M_{3}=\{\alpha_{G}(z):z\in {ℒ}_{G}\setminus {ℒ}_{G}(w),\;\text{LCA}\;\text{of}\;w\;\text{and}\;z\;\text{is}\;\text{tagged}\;\text{asspeciation}\}$*. Let* $M_{w}=(M_{1}|M_{2}|M_{3})$*. Recall* $I_{ij}=|M_{i}\cap P_{j}|$*. The number of SQ quartet equivalence classes anchored to w and mapped from left to the species partition P can be counted as follows:*

(3) $$\begin{array}{cc} & QI_{\mathrm{pro}}(P,M_{w})=|\{\alpha_{G}(Q):Q\subset {ℒ}_{G},Q\overset{w}{\to }P\}|=\\ & \sum_{(i,j,k)\in B_{3},j<k}\left(\begin{array}{c} I_{1i}\\ 2 \end{array}\right)I_{2j}I_{2k}+\sum_{(i,j,k)\in B_{3}}\frac{I_{1i}I_{2j}I_{3k}(I_{1i}+I_{2j}-2)}{2}. \end{array}$$

Lemma 3. *If* $\Omega (G↾Q)≃S↾\alpha_{G}(Q)$*, there exists a unique* P∈P(S)*satisfying* $Q\overset{\psi_{G}(Q)}{\to }P$.

Lemma 4. *Let* $1_{\mathrm{speciation}}(w)$*be 1 for speciation nodes and 0 for duplication nodes and let*

$$w_{\mathrm{pro}}(P)=\sum_{G\in G}\sum_{w\in I(G)}QI_{\mathrm{pro}}(P,M_{w})\times 1_{\mathrm{speciation}}(w)\;.$$

*Then:* $q(S,G)=\sum_{P\in P(S)}w_{\mathrm{pro}}(P)\;.$

Theorem 1. *The ASTRAL-Pro algorithm obtained by replacing W(P) function with* $w_{\mathrm{pro}}(P)$*in the ASTRAL dynamic programing solves the MLQST problem exactly if* $X=2^{S}$.

Proof. By Lemma 4, $\underset{}{\text{arg}{\text{max}}_{S}}q(S,G)=\underset{}{\text{arg}{\text{max}}_{S}}\sum_{P\in P(S)}w_{\text{pro}}(P)$. Thus, ASTRAL dynamic programing can solve the optimization problem exactly given the full search space (the argument is identical to that of ASTRAL and follows from the additive nature of q(S,G)). □

We now make two claims and provide a sketch of proofs in supplementary appendix proofs, Supplementary Material online. Note that by Claim 3, ASTRAL-Pro has polynomial running time.

Claim 2*. For a set of gene trees* G*including only speciations, the tree returned by ASTRAL-Pro is the same as the one returned by ASTRAL.*

Claim3*. The asymptotic running time of ASTRAL-Pro is* $O(D|X{|}^{1.73})=O(D{\left(nN\right)}^{1.73})$*where* $N=\sum_{G\in G}|{ℒ}_{G}|$*and D denotes the number of unique gene tree tripartitions tagged as speciations.*

#### Tagging and Rooting Gene Trees

Gene trees inferred from sequence data are neither rooted nor tagged. We use the heuristics presented in Algorithm 1 to root and tag gene trees, noting that a partially correct rooting suffices (Claim 1). Given a rooted tree, we tag a node as duplication *only if* the node cannot be tagged as speciation by Definition 1 (similar to *observable duplication nodes* defined by Scornavacca et al. [2011]); other nodes are *assumed* to be speciation.

For rooting, we seek the root position that minimizes the number of duplications and losses while allowing for “free” ILS. In more details, in each gene tree *G*, for two nodes *u* and *v* where $\alpha_{G}(u)=\alpha_{G}(v)$, we explain all differences in topologies below *u* and *v* by invoking ILS (as opposed to duplication/loss). Then, three scenarios are possible for a node *u* with children *u_l_* and *u_r_*. 1) When *u* is duplication and $\alpha_{G}(u_{l})=\alpha_{G}(u_{l})$, we do not need to invoke any loss. One duplication suffices. 2) If $\alpha_{G}(u_{l})\subset \alpha_{G}(u_{r})$ or vice versa, we need one loss on *u_l_* and an arbitrary amount of ILS. 3) Else, we need two losses (one in each side) and ILS to describe the differences. Algorithm 1 computes the number of duplication and loss events using this strategy, without penalizing ILS and fixing a cost of one for both duplications and losses. As described, it requires quadratic time per rooting and thus cubic time to find an optimal rooting. In our implementation, we used memoization to reduce this time to quadratic (details omitted). The LCA-based linear algorithm of Scornavacca et al. (2011) could also be adapted.

#### Search Space

We need to constrain the ASTRAL search space to bipartitions in a set *X*. To define *X*, we use a heuristic method relying on several strategies (see Algorithm 2 and Supplementary Material online). First, we use a sampling algorithm (SampleFull procedure) to create single-copy versions of each gene tree, creating a set F. This sampling algorithm prunes the right (or left) subtrees below the highest duplication nodes in the tree, and recurses on each pruned tree, until no species has multiple copies. In addition, per each gene, $2^{C}$ (default: *C *=* *4) single-copy trees are sampled from F, creating a multiset I. This sampling can be probabilistic (taking each side of a duplication with probability 1/2) for high numbers of duplications. When the number of input trees is small, I may become too small; in these cases, I is augmented using another sampling algorithm (SampleExtra procedure). We provide I as input to the algorithms implemented in ASTRAL-III for building the set *X*. Finally, we complete all trees from F using the tree completion algorithm of ASTRAL-III and add the resulting bipartitions to *X*. All methods used guarantee that |X| grows polynomially with the number of species, gene trees, and duplication nodes.

#### Implementation

We implemented Algorithms 1 and 2 as part of a native C++ library called from Java. We based on code on the ASTRAL-MP (Yin et al. 2019) code. The code is available for all platforms, and can exploit multithreading. A-Pro is available at https://github.com/chaoszhang/A-pro. 

#### Statistical Consistency

When the input set G has only speciation nodes, the MLQST problem reduces to the MQSST problem solved by ASTRAL (Mirarab et al. 2014). Thus, like the MQSST, the MLQST is NP-hard (Lafond and Scornavacca 2019). Moreover, the solution to the MQSST problem is a statistically consistent estimator of the species tree under the MSC model and thus ASTRAL-Pro is also statistically consistent in absence of duplication.

In the presence of gene duplication and losses only, let us consider the birth–death model proposed by Arvestad et al. (2009) and refer to it as the GDL model.

Proposition 3. *Under the GDL model, every SQ in every correctly tagged rooted gene tree is isomorphic in topology to the species tree.*

Since all quartets in every equivalence class of SQs match the species tree, the per-locus quartet score will be maximized by the species tree. The following theorem follows.

Theorem 2. *Under the GDL model (Arvestad et al. 2009), the solution to the MLQST problem is a statistically consistent estimator of the species tree for correctly rooted and tagged gene trees.*

In fact, we suspect that ASTRAL-Pro is statistically consistent under the GDL model even when gene trees are imperfectly rooted and tagged. We leave the proof to future work. Finally, note that restricting to *X* does not impact statistical consistency, as each bipartition of the species tree has a nonzero chance of appearing in output of this algorithm.

#### Adopting Local Posterior Probability for A-Pro

By Proposition 3, assuming no error in the input gene trees or their tagging, differences between topologies of SQs and the species tree are due to processes other than GDL. The main such process is ILS. Thus, we can adopt the same quartet-based metric used for measuring support of ASTRAL trees for A-Pro trees.

For each quadripartition A|B|C|D of ${ℒ}_{S}$, representing an internal branch in the species tree, we define $z_{1}$, which is the quartet count of the topology (A∪B)|(C∪D), as

$$\frac{\sum_{G\in G}\sum_{a\in A,b\in B,c\in C,d\in D}|\{\psi_{G}(Q):\alpha_{G}(Q)=ab|cd,Q\in \Sigma_{G}\}|}{\left|A||B||C||D\right|}\;.$$

The quartet counts for (A∪C)|(B∪D) and (A∪D)|(B∪C) are similarly defined and are denoted by $z_{2}$ and $z_{3}$. We use these counts as input the localPP calculation (Sayyari and Mirarab 2016b). Thus,

Definition 8 *.* The localPP support of a branch with counts $z_{1}\ldots \;\;z_{3}$ is defined as

$$\frac{h(z_{1})}{h(z_{1})+2^{z_{2}-z_{1}}h(z_{2})+2^{z_{3}-z_{1}}h(z_{3})},$$

where $h(x)=B(x+1,k\prime -x+2\lambda )(1-I_{\frac{1}{3}}(x+1,k\prime -x+2\lambda ))$, **B** is the beta function, *I_x_* is the regularized incomplete beta function, *λ* is the Yule prior parameter, set by default to 1/2, and $k^{\prime }=z_{1}+z_{2}+z_{3}$.

### Data Sets

We use new and existing simulated data sets as well as a biological data set to test A-Pro.

#### New Simulated Data Set (S25)

We perform a set of simulations using SimPhy (Mallo et al. 2016) starting from a default model condition and adjusting five parameters (table 1). We simulate 50 replicates per condition, and each replicate draws its parameters from prior distributions. Exact commands are given in the Supplementary Material online.

*Default model:* The species tree, simulated under the Yule process with birth rate $5\times 10^{-9}$ and the maximum number of generations of the tree sampled from a log-normal distribution (mean $1.9\times 10^{9}$), has 25 ingroup and an outgroup species. Each replicate has 1,000 true gene trees simulated under DLCoal with fixed haploid population size $N_{e}=4.7\times 10^{8}$. Gene trees have mean ILS level in [60%,80%] range (mean 70%) across replicates (supplementary fig. S2, Supplementary Material online). The duplication rate $\lambda_{+}=4.9\times 10^{-10}$; when there is no loss, gene trees on average include 145 leaves (≈5 extra copies per species). The loss rate $\lambda_{-}$ is set to $\lambda_{+}$; with loss, gene trees have on average 43 leaves. The average number of duplication and loss events are 11 and 9, respectively, but variance is high (supplementary fig. S1, Supplementary Material online). For each gene, we use INDELible (Fletcher and Yang 2009) to simulate gap-free nucleotide sequences along the gene trees using the GTR + Γ model (Tavaré 1986) with two different sequence lengths: 500 and 100 bp. We then use FastTree2 (Price et al. 2010) to estimate maximum likelihood gene trees under the GTR + Γ model. Gene tree estimation error, measured by the false negative rate between the true gene trees and the estimated gene trees, depends on the sequence length and fluctuates significantly (from 0% to 100%) both within and across replicates (supplementary fig. S3, Supplementary Material online); mean error is 36% and 15% for 100 and 500 bp, respectively.

*Controlling* $\lambda_{+},\lambda_{-}$: Here, we consider 5 × 4 = 20 conditions, changing duplication and loss rates. Our $\lambda_{+}$ settings result in 0–5 extra copies per gene, and the $\lambda_{-}/\lambda_{+}$ varies between 0 and 1 (table 1 and supplementary fig. S4, Supplementary Material online). All other parameters are identical to the default condition.

*Controlling* $\lambda_{+},N_{e}$: Here, we consider 3 × 5 = 15 conditions, fixing $\lambda_{-}$ to be equal to $\lambda_{+}$, but changing $\lambda_{+}$ and ILS levels (controlled by *N*_e_). Our $\lambda_{+}$ settings result in 0–5 extra copies per gene, and the mean ILS level between true and estimated gene trees varies between 0% and 70% RF. (table 1 and supplementary fig. S5, Supplementary Material online) All other parameters are identical to the default model.

*Controlling n*: Fixing all parameters, we vary the number of ingroup taxa *n* from 10 to 500.

*Controlling k*: Fixing all parameters, we vary the number of gene trees *k* from 25 to 10,000.

#### Existing Simulations (S100)

We also used an existing data set that Molloy and Warnow (2019) simulated based on a real fungal data set (Rasmussen and Kellis 2012). The simulation protocol of this data set is similar to that of S25 data set, with some notable differences. 1) The data set included 100 species (no outgroup); species tree height, speciation rate, and mutation rates all differed from S25. 2) Shorter gene alignments were also used, resulting in higher MGTE (25 bp: 67%, 50 bp: 52%, 100 bp: 35%, and 500 bp: 19%). 3) The duplication rate $\lambda_{+}$ was set to $1\times 10^{-10},\;2\times 10^{-10}$, or $5\times 10^{-10}$ (named 1, 2, and 5, respectively), and the duplication rate equaled the loss rate for all model conditions. 4) ILS was much lower than S25; two conditions were simulated with *N*_e_ set to $1\times 10^{7}$ and $5\times 10^{7}$ (named 1 and 5, respectively), which result in 2% and 12% RF between true gene trees and the species tree. 5) Gene trees were estimated using RAxML instead of FastTree2.

#### Biological Data

Wickett et al. (2014) have performed a transcriptome analysis of 103 plant species and 424 single-copy gene trees (out of thousands of genes) using both concatenation and ASTRAL. In preliminary analyses, the authors had inferred multicopy gene trees using RAxML from 9,683 genes for 83 of those species, ranging in size between 5 and 2,395 leaves. However, not being able to obtain an accurate species tree from the multicopy gene trees, they abandoned the strategy in later analyses. The gene trees are available from Matasci et al. (2014). We used RAxML gene trees inferred from the first two codon positions (C12) as the original study.

For the fungal data set, all the peptide ML gene trees were downloaded from Butler et al. (2009) and used here. We used peptide gene trees because the reference species tree, inferred through concatenation using MrBayes (Huelsenbeck and Ronquist 2001), also uses peptide sequences. Authors comment on unreliability of their nucleotide-based analyses due to grouping by GC content.

### Methods Compared

We compare A-Pro with the following methods, which are the leading methods that can handle multiple copies. Another method, STAG (Emms et al. 2018), is not included because of its poor performance in the study by Molloy and Warnow (2019), including that it fails to run on some model conditions (supplementary fig. S11, Supplementary Material online).

DupTree (Wehe et al. 2008) infers a species tree from rooted or unrooted gene trees minimizing the duplication reconciliation cost (Maddison 1997) under the duplication-only model, but it does not model ILS. We provide DupTree with unrooted gene trees. We also tried iGTP, minimizing DupLoss score, but we only show results in supplementary figure S7, Supplementary Material online, as it was almost universally worse than DupTree.

MulRF (Chaudhary et al. 2013), based on an extension of the RF distance (Robinson and Foulds 1981) to multilabeled trees, is a hill-climbing method that aims at finding the tree with the minimum RF distance to the input. We use MulRF because of its advantage over other methods shown in previous studies (Chaudhary et al. 2015).

ASTRAL-multi (Rabiee et al. 2019) is a feature of ASTRAL designed for handling multiple individuals. Legried et al. (2020) propose to use ASTRAL-multi for multicopy data. Due to its high memory requirements, we were able to include it in only one experiment of S25.

## Data Availability

The code is available at https://github.com/chaoszhang/A-pro (doi:10.5281/zenodo.3858153) and data are made available at https://github.com/chaoszhang/A-pro_data (doi:10.5281/zenodo.3858155).

## Supplementary Material

Supplementary data are available at *Molecular Biology and Evolution* online.

## Supplementary Material

msaa139_Supplementary_Data
