# Corrigendum to: ASTRAL-Pro: Quartet-Based Species-Tree Inference despite Paralogy

**DOI:** 10.1093/molbev/msab232

**Published:** 2021-08-21

**Authors:** Chao Zhang, Celine Scornavacca, Erin K Molloy, Siavash Mirarab

*Molecular Biology and Evolution*, Volume 37, Issue 11, November 2020, Pages 3292–3307, https://doi.org/10.1093/molbev/msaa139

In the originally published version of this manuscript, there was an error in Definition 1 which affected the Proof of Proposition 1 in the supplementary material.

The correct text of Definition 1 should read:

“Definition 1 (Tagged trees). We say that a rooted tree *G* is tagged if every internal node is tagged either as duplication or as speciation. A node *u* with children $u_{1}$ and $u_{2}$ can be tagged as speciation only if the sets $\alpha_{G}\left(u_{1}\right)$ and $\alpha_{G}\left(u_{2}\right)$ are mutually exclusive.”

Definition 1 and the Proof of Proposition 1 in the supplementary material have been corrected online.

