# Supplementary material for: ASTRAL-Pro: Quartet-Based Species-Tree Inference despite Paralogy
Source: Mol Biol Evol. 2020 Sep 4;37(11):3292–307. doi: 10.1093/molbev/msaa139 (PMC7751180; doi:10.1093/molbev/msaa139)
Supplement: msaa139_Supplementary_Data [file msaa139_supplementary_data.pdf]

Online supplementary material

ASTRAL-Pro: Quartet-based Species Tree Inference Despite Paralogy

Proofs

PROOF OF PROPOSITION 1. Denote $Q_1 = \{a,b,c,d\}$ and $Q_2 = \{\tilde{a},\tilde{b},\tilde{c},\tilde{d}\}$ (with obvious correspondence of labels). Let w be the anchor LCA and note that anchor LCA is the LCA of three (if $Q_1 \angle G$) or four (if $Q_1 \perp G$) of the quartet leaves; thus, by Definition 2, w is a speciation node or otherwise Q_1 would not be a SQ. Let the children of w be denoted by w_1 and w_2; by Definition 1, $\alpha_G(w_1)$ and $\alpha_G(w_2)$ must be mutually exclusive. In the unbalanced case, w.l.o.g, assume the topology is $(((a,b),c),d)$; then, let u denote the LCA of w and d and note that u is the LCA of a, b, and d. Thus, by Definition 2, u is a speciation node or otherwise Q_1 would not be a SQ. Let the children of u be denoted by u_1 and u_2, and w.l.o.g., let u_1 be the child on the same side as w. By Definition 1, $\alpha_G(u_1)$ and $\alpha_G(u_2)$ must be mutually exclusive. Therefore $\alpha_G(w) \subseteq \alpha_G(u_1)$. Consequently, $\alpha_G(w_1)$, $\alpha_G(w_2)$, and $\alpha_G(u_2)$ are mutually exclusive. Given that $a,b \in \mathcal{L}_G(w_1)$, $c \in \mathcal{L}_G(w_2)$, $d \in \mathcal{L}_G(u_2)$, mutual exclusivity is possible only if $\tilde{a},\tilde{b} \in \mathcal{L}_G(w_1)$, $\tilde{c} \in \mathcal{L}_G(w_2)$, $\tilde{d} \in \mathcal{L}_G \setminus \mathcal{L}_G(u_1)$. In the case of balanced topology (w.l.o.g, $((a,b),(c,d))$), mutual exclusivity of $\alpha_G(w_1)$ and $\alpha_G(w_2)$ and the fact that $a,b \in \mathcal{L}_G(w_1)$ and $c,d \in \mathcal{L}_G(w_2)$ implies that $\tilde{a},\tilde{b} \in \mathcal{L}_G(w_1)$, $\tilde{c},\tilde{d} \in \mathcal{L}_G(w_2)$. Thus, in either case, $\Omega(G \upharpoonright Q_1) \simeq \Omega(G \upharpoonright Q_2)$. □

PROOF OF PROPOSITION 2. Each node of a gene tree represents an ancestral or present-day gene and thus belongs to a locus. The children of a speciation node stay in the same locus that their parent, while for a duplication node we have that exactly one of the two children change locus and the other stays in the same locus than its parent. Therefore, all nodes under w, which is a speciation node, belong to the descendants (including itself) of the locus to which w belongs, and when tracing back to the time of speciation event w, they will lead to the same locus. Since all equivalence classes share the same anchor LCA, the result follows. □

PROOF OF LEMMA 1. Note that P can anchor Q_1 only iff any species tree that includes P must match the gene tree topology for Q_1 By Proposition 1, due to equivalence of Q_1 and Q_2, we infer Q_2 must (i) match the same species quartet set as Q_1 and (ii) share the same anchor LCA w. Thus, P can also anchor Q_2. (iii) When $Q_1 \angle G$ as shown in Figure 7, $\tilde{a},\tilde{b} \in \mathcal{L}_G(w_1)$ are the leaves mapped to the quartet tree and thus mapped to the same partition as a,b; similarly, when $Q_1 \perp G$, the pair of leaves under left subtree of the anchor LCA of both quartets map to the same partition of P. □

Proof of Lemma 2. First note that:

$$|\{\alpha_G(Q): Q\subset \mathcal{L}_G, Q \xrightarrow{w} P\}| =$$
$$|\{\alpha_G(Q): Q\subset \mathcal{L}_G, Q \xrightarrow{w} P, Q \perp G\}| +$$
$$|\{\alpha_G(Q): Q\subset \mathcal{L}_G, Q \xrightarrow{w} P, Q \angle G\}|$$

We compute each part separately. Recall here that, since $Q \xrightarrow{w} P$, Q is a SQ quartet and thus $|Q| = |\alpha_G(Q)| = 4$.

When $Q \perp G$, let $Q = \{a,b,c,d\}$ with $\alpha_G(a), \alpha_G(b) \in M_1, \alpha_G(c), \alpha_G(d) \in M_2$. Since $Q \xrightarrow{w} P$, leaves $\alpha_G(a)$ and $\alpha_G(b)$ must be in the same partition of P. When $\alpha_G(a), \alpha_G(b) \in P_1$, leaves $\alpha_G(c)$ and $\alpha_G(d)$ must be in partition P_2 and P_3 respectively since P can anchor Q. W.l.o.g., we can assume $\alpha_G(c) \in P_2$. Therefore, $\alpha_G(a), \alpha_G(b) \in M_1 \cap P_1$, $\alpha_G(c) \in M_2 \cap P_2$, $\alpha_G(d) \in M_2 \cap P_3$. The number of such $\alpha_G(Q)$ is $\binom{|M_1\cap P_1|}{2}|M_2 \cap P_2||M_2 \cap P_3| = \binom{I_{11}}{2} I_{22} I_{23}$. Similarly when $\alpha_G(a), \alpha_G(b) \in M_1 \cap P_2$ and $\alpha_G(a), \alpha_G(b) \in M_1 \cap P_3$, the number of such $\alpha_G(Q)$ is $\binom{I_{12}}{2} I_{21} I_{23}$ and $\binom{I_{13}}{2} I_{21} I_{22}$ respectively. Thus,

$$|\{\alpha_G(Q): Q\subset \mathcal{L}_G, Q \xrightarrow{w} P, Q \perp G\}| = \binom{I_{11}}{2} I_{22} I_{23} + \binom{I_{12}}{2} I_{21} I_{23} + \binom{I_{13}}{2} I_{21} I_{22}$$

Similarly, when $Q \angle G$, let $Q = \{a,b,c,d\}$ with $\alpha_G(a)$ and $\alpha_G(b)$ in the same partition of P. Notice that, in the unbalanced case, $\alpha_G(a)$ and $\alpha_G(b)$ can be both either in M_1 or either in M_2, and since c and d are not interchangeable as in the balanced case, we can have $\alpha_G(a), \alpha_G(b) \in P_i, \alpha_G(c) \in P_j, \alpha_G(d) \in P_k$ for (i,j,k) with any permutation of $(1,2,3)$, from the definition of P anchoring Q. All together we have 12 cases.

In the case that $\alpha_G(a), \alpha_G(b) \in P_1, \alpha_G(c) \in P_2, \alpha_G(d) \in P_3$, and $\alpha_G(a), \alpha_G(b) \in M_1$, we have $\alpha_G(a), \alpha_G(b) \in M_1 \cap P_1$, $\alpha_G(c) \in M_2 \cap P_2$, and $\alpha_G(d) \in M_3 \cap P_3$. The number of such $\alpha_G(Q)$ is $\binom{|M_1\cap P_1|}{2}|M_2 \cap P_2||M_3 \cap P_3| = \binom{I_{11}}{2} I_{22} I_{33}$. The other 11 permutations are similar. In total,

$$\begin{aligned}
&|\{\alpha_G(Q): Q\subset \mathcal{L}_G, Q \xrightarrow{w} P, Q \angle G\}| \\
=&\binom{I_{11}}{2}(I_{22}I_{33} + I_{32}I_{23}) + \binom{I_{12}}{2}(I_{21}I_{33} + I_{31}I_{23}) \\
&+\binom{I_{13}}{2}(I_{21}I_{32} + I_{31}I_{22}) + \binom{I_{21}}{2}(I_{12}I_{33} + I_{32}I_{13}) \\
&+\binom{I_{22}}{2}(I_{11}I_{33} + I_{31}I_{13}) + \binom{I_{23}}{2}(I_{11}I_{32} + I_{31}I_{12})
\end{aligned} \tag{S1}$$

Thus,

$$
\begin{aligned}
QI_{pro}(P,M_w) = &|\{\alpha_G(Q): Q \subset \mathcal{L}_G, Q \xrightarrow{w} P\}| = \\
&\binom{I_{11}}{2} I_{22}I_{23} + \binom{I_{12}}{2} I_{21}I_{23} + \binom{I_{13}}{2} I_{21}I_{22} \\
&+ \binom{I_{11}}{2}(I_{22}I_{33}+I_{32}I_{23}) + \binom{I_{12}}{2}(I_{21}I_{33}+I_{31}I_{23}) + \binom{I_{13}}{2}(I_{21}I_{32}+I_{31}I_{22}) \\
&+ \binom{I_{21}}{2}(I_{12}I_{33}+I_{32}I_{13}) + \binom{I_{22}}{2}(I_{11}I_{33}+I_{31}I_{13}) + \binom{I_{23}}{2}(I_{11}I_{32}+I_{31}I_{12})
\end{aligned}
\tag{S2}
$$

With simple manipulations, it can be shown that the right hand side of this equation can be rewritten as:

$$
\sum_{(i,j,k)\in B_3, j<k} \binom{I_{1i}}{2} I_{2j}I_{2k} + \sum_{(i,j,k)\in B_3} \frac{I_{1i}I_{2j}I_{3k}(I_{1i}+I_{2j}-2)}{2}
$$

□

Proof of Lemma 3. Let $\Omega(G \upharpoonright Q)$ be designated by $ab|cd$ and assume w.l.o.g that the anchor corresponding to a and b is the first anchor observed on the post-order traverse of G. It is easy to show (see Mirarab *et al.* (2014)) that if $\Omega(G \upharpoonright Q) \simeq S \upharpoonright \alpha_G(Q)$ there exist exactly two tripartitions P^1 and P^2 in $\mathcal{P}(S)$ that imply a quartet topology that matches $\Omega(G \upharpoonright Q)$ (condition (*ii*) of Definition 7). Each of the two tripartitions has two leaves of $\alpha_G(Q)$ in one of its parts and the other two leaves fall on two different parts. Also, the two leaves that are together can only be a and b or c and d and thus, only one of P^1 and P^2 would include both a and b in the same part. Therefore, by condition (*iii*) of Definition 7, exactly one of $Q \xrightarrow{\psi_G(Q)} P^1$ and $Q \xrightarrow{\psi_G(Q)} P^2$ can be true. □

Proof of Lemma 4.

$$
\begin{aligned}
q(S,\mathcal{G}) &= \sum_{G\in\mathcal{G}} q(S,G) && \text{(S3)}\\
&= \sum_{G\in\mathcal{G}} |\{(\alpha_G(Q),\psi_G(Q)) : Q \in \Sigma_G, \Omega(G \upharpoonright Q) \simeq S \upharpoonright \alpha_G(Q)\}| && \text{(S4)}\\
&= \sum_{P\in\mathcal{P}(S)} \sum_{G\in\mathcal{G}} |\{(\alpha_G(Q),\psi_G(Q)) : Q \subset \mathcal{L}_G, Q \xrightarrow{\psi_G(Q)} P\}| && \text{(S5)}\\
&= \sum_{P\in\mathcal{P}(S)} \sum_{G\in\mathcal{G}} \sum_{w\in I(G)} |\{\alpha_G(Q) : Q \subset \mathcal{L}_G, Q \xrightarrow{w} P\}| && \text{(S6)}\\
&= \sum_{P\in\mathcal{P}(S)} \sum_{G\in\mathcal{G}} \sum_{w\in I(G)} |\{\alpha_G(Q) : Q \subset \mathcal{L}_G, Q \xrightarrow{w} P\}| \times \mathbf{1}_{speciation}(w) && \text{(S7)}\\
&= \sum_{P\in\mathcal{P}(S)} \sum_{G\in\mathcal{G}} \sum_{w\in I(G)} QI_{pro}(P,M_w) \times \mathbf{1}_{speciation}(w) && \text{(S8)}\\
&= \sum_{P\in\mathcal{P}(S)} w_{pro}(P) && \text{(S9)}
\end{aligned}
$$

The first two lines are implied by Definition 5. Equation (S5) follows from Lemma 1 and Lemma 3 that together establish that each equivalence class of quartets maps to exactly one P. Equation (S6) follows from Definition 4 combined with a simple rearrangement obtained by counting unique tuples once. Equation (S7) follows from the fact that when w is a duplication node, $|\{\alpha_G(Q)\colon Q \subset \mathcal{L}_G, Q \xrightarrow{w} P\}| = 0$. Equation (S8) follows from Lemma 2. □

Proof sketch of Claim 1. The rooting that minimizes the number of duplications and losses (#duploss for short) in Alg. 1 may not be unique. In particular, if a rooted tree G minimizes #duploss, then rooting it at any branch such that the path between the parent node of the branch and the current root (including the two end nodes) does not contain any duplication node will also minimize #duploss. We call a correctly-tagged gene tree partially-correctly-rooted if the path between the parent node of the branch where it is rooted and the root in the correctly-rooted tree does not contain any duplication node. In particular, when gene trees do not have duplications, then any rooting of a gene tree is partially-correctly. We observe that the equivalence classes of quartets in all partially-correctly-rooted trees stay the same (although *all* quartet trees in the same equivalence class may change from balanced to unbalanced or vice versa), and thus any partially-correct-rooting of gene trees will result in the same species tree. □

Sketch of proof of Claim 2. When $\mathcal{G}$ only includes speciation nodes, regardless of rooting, each quartet is a SQ. Since each leaf corresponds to distinct taxa in the species tree, each quartet equivalence class contains only one quartet. Therefore, each quartet is counted exactly once and thus $\sum_{P \in \mathcal{P}(S)} w_{pro}(P) = \sum_{P \in \mathcal{P}(S)} W(P)$ regardless of rooting. □

Sketch of proof of Claim 3 (Running time of ASTRAL-Pro). Let

$$N = \sum_{G \in \mathcal{G}} |\mathcal{L}_G|$$

denote the sum of the number of leaves in the gene trees. Then the number of anchor LCAs in all gene trees is $O(N)$. Let D denote the number of unique gene tree tripartitions tagged as speciations and note $D = O(N)$. By only counting each unique gene tree tripartition once against each species tree tripartition, the running time of ASTRAL-Pro becomes $O(D|X|^{1.73})$ (by an argument that is identical to that provided for ASTRAL-III (Mirarab *et al.*, 2014) and follows from results of Kane and Tao (2017). However, while ASTRAL-III guarantees $|X| = O(nk)$ with $k = |\mathcal{G}|$, in ASTRAL-Pro, in the presence of duplications, $|X|$ can be large; in particular with our sampling algorithm (Algorithm 2), $|X| = O(nN)$. Thus, the running time of A-Pro is $O(D(nN)^{1.73})$. Note that this analysis is not tight and can be made more precise in the future. Also, in the future, we will explore sub-sampling a constant number of trees

from the output of Algorithm 2 per gene tree, which will limit the $|X|=nk$ and thus limit the running time of ASTRAL-pro to $O(D(nk)^{1.73})$. □

PROOF OF PROPOSITION 3. Under GDL, besides leaves, each internal node $u_G \in I(G)$ in a gene tree G corresponds to an internal node $u_S \in I(S)$; if u_G is a duplication node, u_S is the node down the branch in S where the duplication event happened, and if u_G is a speciation node, u_S is the respective speciation node. It is easy to see that $\alpha_G(u_G) \subset \mathcal{L}_S(u_S)$. For each SQ quartet $Q=\{a,b,c,d\}$, assuming w.o.l.g that $G \upharpoonright Q$ has unrooted topology $ab|cd$, let $w_G=\psi_G(Q)$, and u_G and v_G be the children of w_G. Let u_G, v_G, and w_G correspond to u_S, v_S, and w_S in S, respectively. Since w_G is a correctly tagged speciation node, u_S and v_S are descendants from different children of w_S.

When $Q \perp G$, assuming w.o.l.g. $a,b \in \mathcal{L}_G(u_G)$ and $c,d \in \mathcal{L}_G(v_G)$, we get $\alpha_G(a),\alpha_G(b) \in \mathcal{L}_S(u_S)$ and $\alpha_G(c),\alpha_G(d) \in \mathcal{L}_S(v_S)$ and thus $\alpha_G(a)\alpha_G(b)|\alpha_G(c)\alpha_G(d)$ is induced by S.

When $Q \angle G$, assuming w.o.l.g. $a,b \in \mathcal{L}_G(u_G)$, $c \in \mathcal{L}_G(v_G)$, and $d \notin \mathcal{L}_G(w_G)$, we get $\alpha_G(a),\alpha_G(b) \in \mathcal{L}_S(u_S)$ and $\alpha_G(c) \in \mathcal{L}_S(v_S)$. Since d is not under w_G, $\alpha_G(d)$ and w_S are under different children of the species tree node to which the LCA of d and w_G corresponds. Therefore, $\alpha_G(d) \notin \mathcal{L}_S(w_S)$ and thus $\alpha_G(d) \notin \mathcal{L}_S(u_S)$; since $\alpha_G(a) \in \mathcal{L}_S(u_S)$ and $\alpha_G(b) \in \mathcal{L}_S(u_S)$, it follows that $\alpha_G(a)\alpha_G(b)|\alpha_G(c)\alpha_G(d)$ in S. □

Supplementary Algorithms

Algorithm 2 Building set X. Default constant parameters: $C=4$, $E_m=500$, $E_s=4$. The algorithm uses the (arbitrary) left/right orientation of children of a node as given in the input.

procedure BUILDX($\mathcal{G}$)
  $\mathcal{F}=\emptyset$ and $\mathcal{I}=\emptyset$
  for $G \leftarrow \mathcal{G}$ **do**
    $(M,S) \leftarrow$ SAMPLEFULL($G,\mathcal{L}_G,C$)
    $\mathcal{F} \leftarrow \mathcal{F} \cup S$
    $\mathcal{I} \leftarrow \mathcal{I} \uplus M$
  for $G \in \{$randomly sample $\max(0,\min(|\mathcal{G}|,\frac{E_m-|\mathcal{G}|}{E_s}))$ trees from $\mathcal{G}\}$ **do**
    $\mathcal{I} \leftarrow \mathcal{I} \uplus$ SAMPLEEXTRA(G, $\mathcal{L}_G$)
  $X \leftarrow$ run all ASTRAL-III methods for building X with $\mathcal{I}$ as input (i.e., `-i` $\mathcal{I}$)
  $X \leftarrow X \cup \Big($all bipartitions of $\{G$ completed via the ASTRAL-III tree-completion method $\forall G \in \mathcal{F}\}\Big)$
procedure SAMPLEFULL(G, A, c)
  if $|\alpha_G(A)|=|A|$ **then**
    return (multiset: $[\Omega(G \restriction A)$ repeated 2^c times$]$, set: $\{\Omega(G \restriction A)\}$)
  else
    $A_l \leftarrow \emptyset$ and $A_r \leftarrow \emptyset$
    $G_A \leftarrow G \restriction A$ (degree-2 nodes removed)
    for $a \in A$ **do**
      $p \leftarrow$ the highest ancestor of a in G_A tagged as a duplication node (or $\emptyset$ if it doesn't exist)
      if $(p=\emptyset) \vee (a$ is to the left of $p)$ **then**
        $A_l \leftarrow A_l \cup \{a\}$
      if $(p=\emptyset) \vee (a$ is to the right of $p)$ **then**
        $A_r \leftarrow A_r \cup \{a\}$
    $(L.m,L.s) \leftarrow$ SAMPLEFULL($G,A_l,\max(c-1,0)$)
    $(R.m,R.s) \leftarrow$ SAMPLEFULL($G,A_r,\max(c-1,0)$)
    if $c=0$ **then**
      return (multiset: randomly select $L.m$ or $R.m$ with equal probabilities, set: $L.s \cup R.s$)
    else
      return (multiset: $L.m \uplus R.m$, set: $L.s \cup R.s$)
procedure SAMPLEEXTRA(G, A)
  if $|\alpha_G(A)|=|A|$ **then**
    return multiset $[\Omega(G)$ repeated once$]$
  else
    $A_l \leftarrow \emptyset$ and $A_r \leftarrow \emptyset$
    $G_A \leftarrow G \restriction A$ (degree-2 nodes removed)
    for $a \in A$ **do**
      $p \leftarrow$ the highest ancestor of a in G_A tagged as a duplication node (or $\emptyset$ if it doesn't exist)
      if $(p \neq \emptyset) \wedge (a$ is to the left of $p)$ **then**
        $A_l \leftarrow A_l \cup \{a\}$
      if $(p \neq \emptyset) \wedge (a$ is to the right of $p)$ **then**
        $A_r \leftarrow A_r \cup \{a\}$
    $B_l \leftarrow \{x : x \in A_l, \alpha_G(x) \in \alpha_G(A_l) \setminus \alpha_G(A_r)\}$
    $B_r \leftarrow \{x : x \in A_r, \alpha_G(x) \in \alpha_G(A_r) \setminus \alpha_G(A_l)\}$
    $G_L \leftarrow G \restriction ((\mathcal{L}_G \setminus A_r) \cup B_r)$ (degree-2 nodes removed)
    $G_R \leftarrow G \restriction ((\mathcal{L}_G \setminus A_l) \cup B_l)$ (degree-2 nodes removed)
    for internal node u of G_L where $\mathcal{L}_G(u) \subset B_r$ **do**
      $B_u \leftarrow \{$one leaf node arbitrarily chosen from $\{x : \alpha_G(x)=s, x \in \mathcal{L}_G(u)\} : s \in \alpha_G(u)\}$
      replace u with a star tree consisting of leaves from the set B_u
    for internal node u of G_R where $\mathcal{L}_G(u) \subset B_l$ **do**
      $B_u \leftarrow \{$one leaf node arbitrarily chosen from $\{x : \alpha_G(x)=s, x \in \mathcal{L}_G(u)\} : s \in \alpha_G(u)\}$
      replace u with a star tree consisting of leaves from the set B_u
    $R=$ SAMPLEEXTRA(G_L,A_l) $\uplus$ SAMPLEEXTRA(G_R,A_r)
    return R

Simulation details

Simphy command for default parameters:

```
simphy -sl f:25 -rs 50 -rl f:1000 -rg 1 -sb f:0.000000005 -sd f:0
-st ln:21.25,0.2 -so f:1 -si f:1 -sp f:470000000 -su ln:-21.9,0.1
-hh f:1 -hs ln:1.5,1 -hl ln:1.551533,0.6931472 -hg ln:1.5,1 -cs 9644
-v 3 -o default -ot 0 -op 1 -lb f:0.00000000049 -ld f:0.00000000049
-lt f:0
```

Other settings use a similar command with parameters changed according to the table below.

Table S1. Simphy parameters for all experiments

**Parameter name**	**Parameter value**
Default Parameters	
Speciation rate	5e-9
Extinction rate	0
Locus trees	1000
Gene trees	1
Number of leaves	25 + an outgroup
Ingroup divergence to the ingroup ratio	1.0
Generations	LogN(21.25,0.2)
Haploid effective population size	4.7e+8
Global substitution rate	LogN(-21.9,0.1)
Lineage specific rate gamma shape	LogN(1.5,1)
Gene family specific rate gamma shape	LogN(1.551533,0.6931472)
Gene tree branch specific rate gamma shape	LogN(1.5,1)
Duplication rate	4.9e-10
Loss rate to duplication rate ratio	1
Seed	9644
Sequence length	500, 100
Sequence base frequencies	Dirichlet(A=36,C=26,G=28,T=32)
Sequence transition rates	Dirichlet(TC=16,TA=3,TG=5,CA=5,CG=6,AG=15)
Controlling Duplication and Loss Rates (5×4 conditions)	
Duplication rate	4.9e-10, 2.7e-10, 1.9e-10, 5.2e-11, 0
Loss rate to duplication rate ratio	1, 0.5, 0.1, 0
Controlling Duplicatoin and ILS Rate (3×4 conditions)	
Duplication rate	4.9e-10, 1.9e-10, 0
Haploid effective population size	4.7e+8, 1.9e+8, 4.8e+7, 1e+4
Controlling n	
Number of leaves	10, 25, 100, 250, 500 + an outgroup
Controlling k	
Locus trees	25, 100, 250, 1000, 2500, 10000

Supplementary Figures and Tables

	1st	2nd	3rd	4th
MulRF	42	67	10	1
DupTree	28	8	15	69
A-Pro	105	14	1	0
ASTRAL-multi	12	14	71	23

Table S2. Rank of methods on S100 dataset over all 120 test conditions. Ranks are obtained using mean species tree error, rounded to two significant digits to create tie for cases where error values are extremely close.

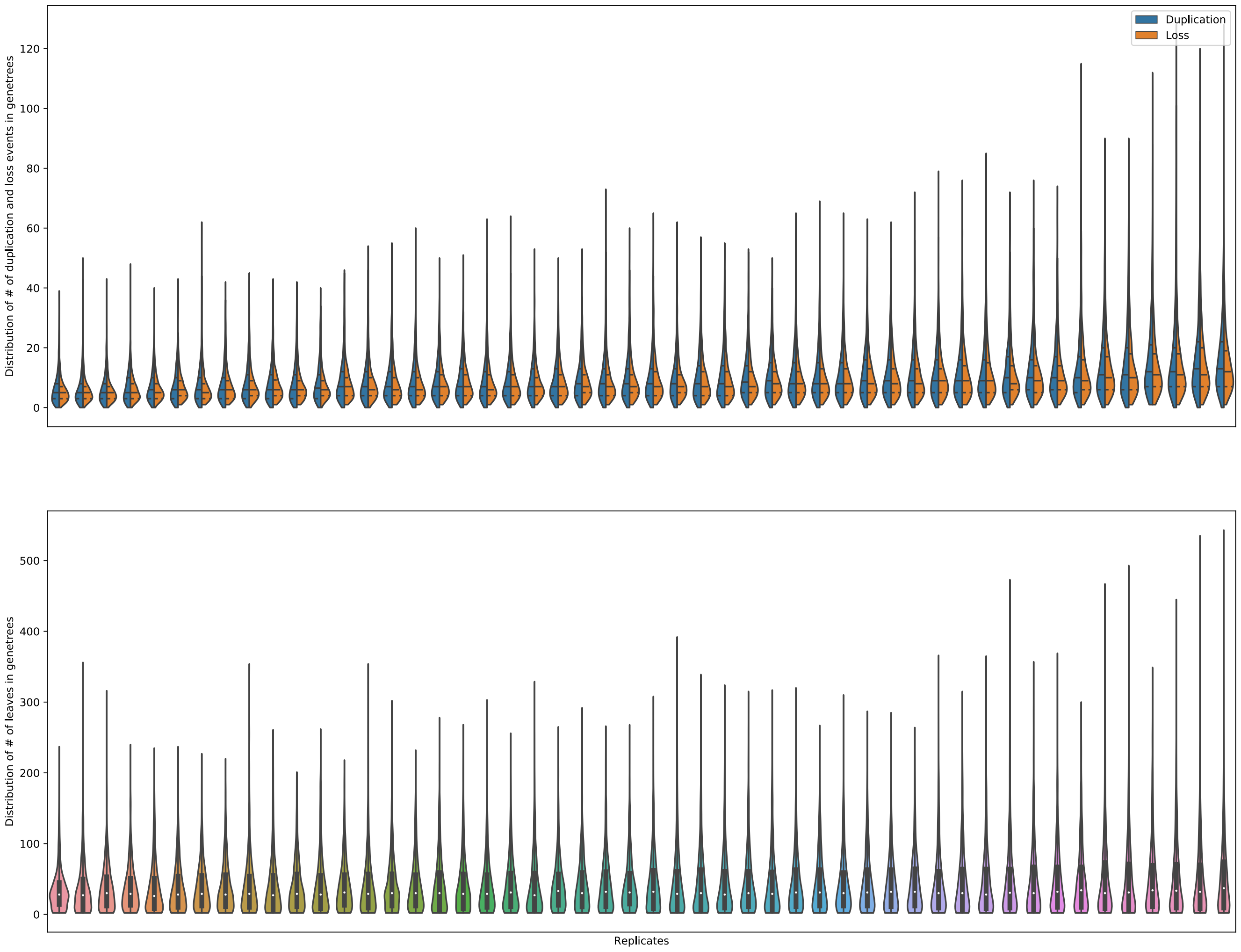

FIG. S1. Distribution of the number of duplication events, loss events and sizes of leaf set for gene trees in the default condition by replicates. The figure on the top is sorted by the mean number of duplication events and the figure on the bottom is sorted by mean leaf set size.

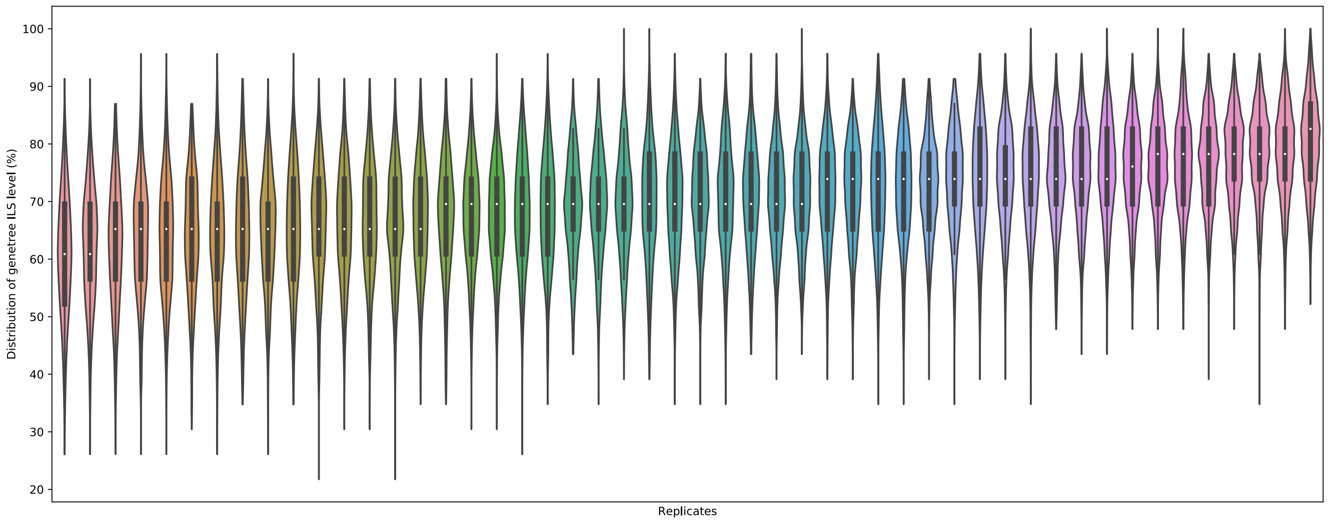

FIG. S2. Distribution of genetree ILS, as measured by the normalized RF distance between true gene trees and the true species, in the condition with all default parameters but $\lambda_+ = \lambda_- = 0$. Results are divided by replicates, sorted by mean ILS level.

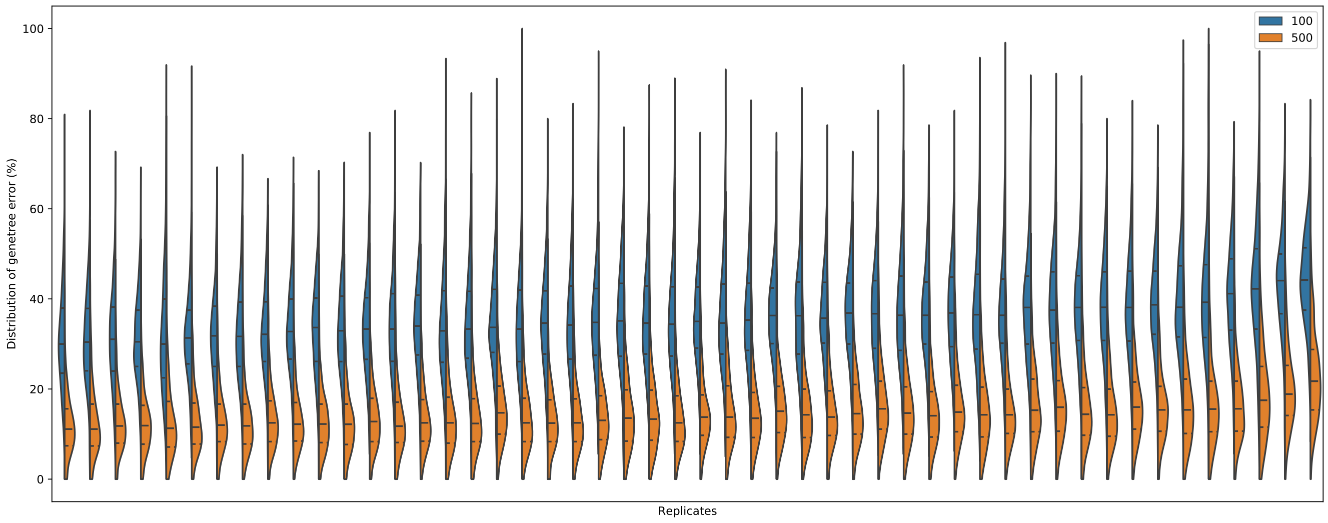

FIG. S3. Distribution of the gene tree errors (normalized RF distance between true gene trees and the estimated gene tree) for inferred trees with at least 14 leaves in the default condition. Results are divided by sequence length (100bps or 500bps) and by replicates, sorted by mean gene tree error of the 100bps condition.

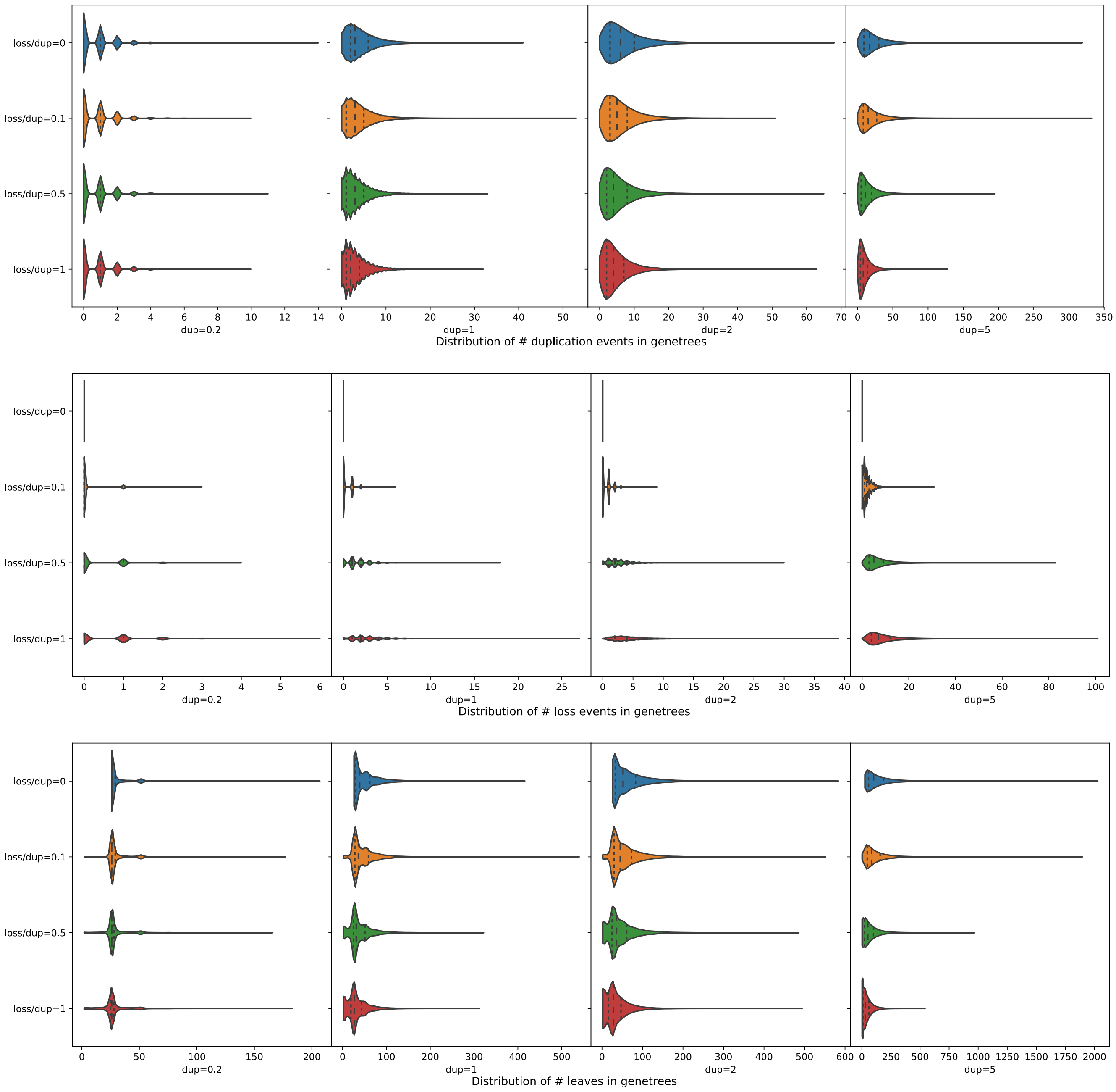

FIG. S4. Distribution of the number of duplication events, loss events and sizes of leaf set for gene trees of each replicates sorted by duplication and loss rate.

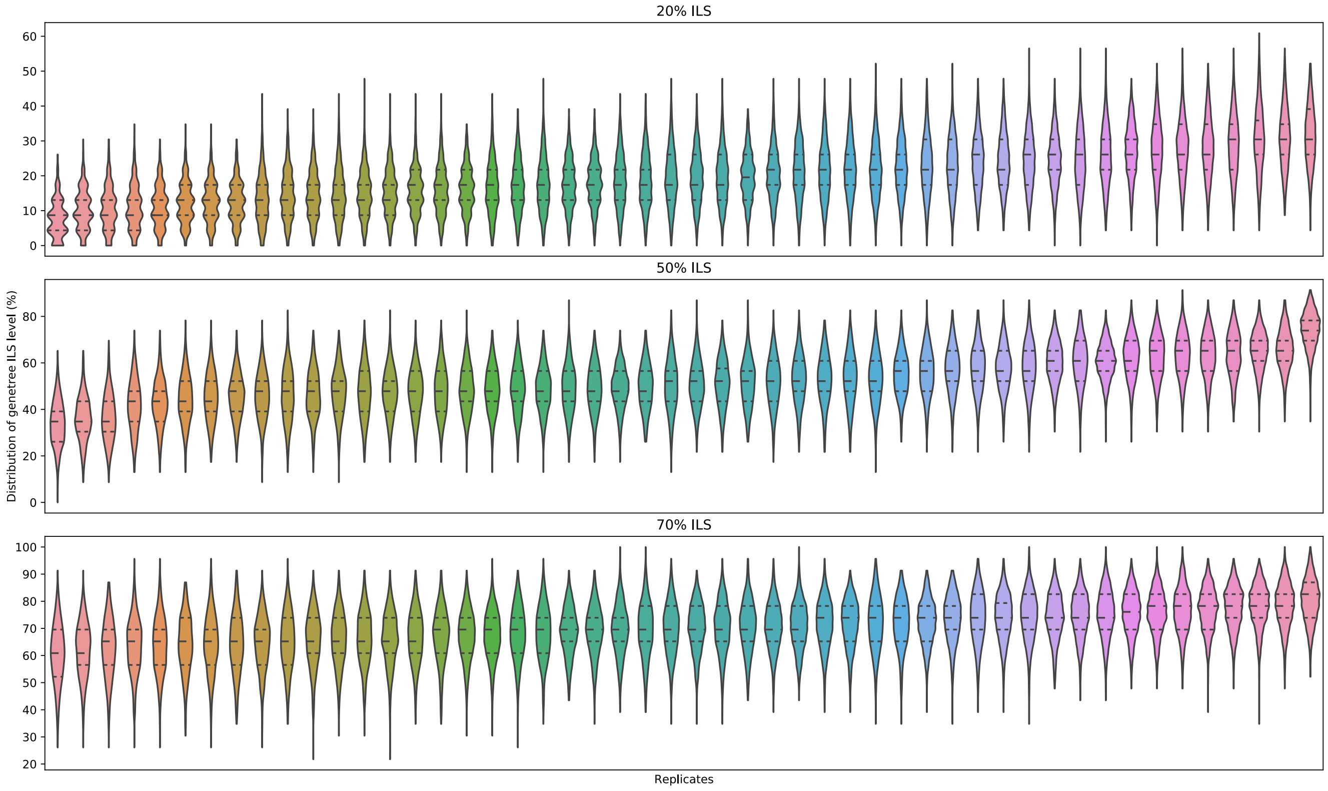

FIG. S5. Distribution of gene tree ILS levels by replicates and expected ILS level, sorted by mean ILS level.

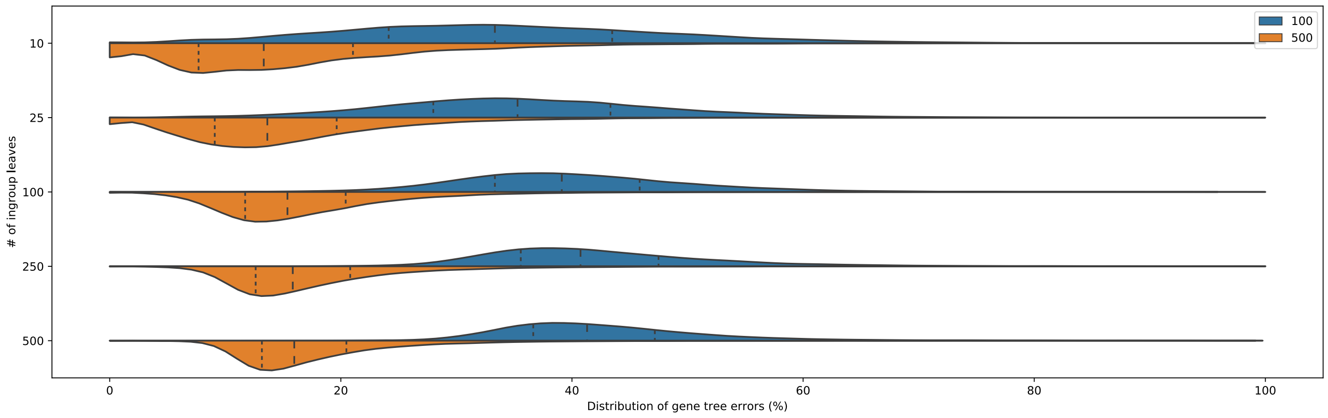

FIG. S6. Distribution of gene tree errors by the number of ingroup species n.

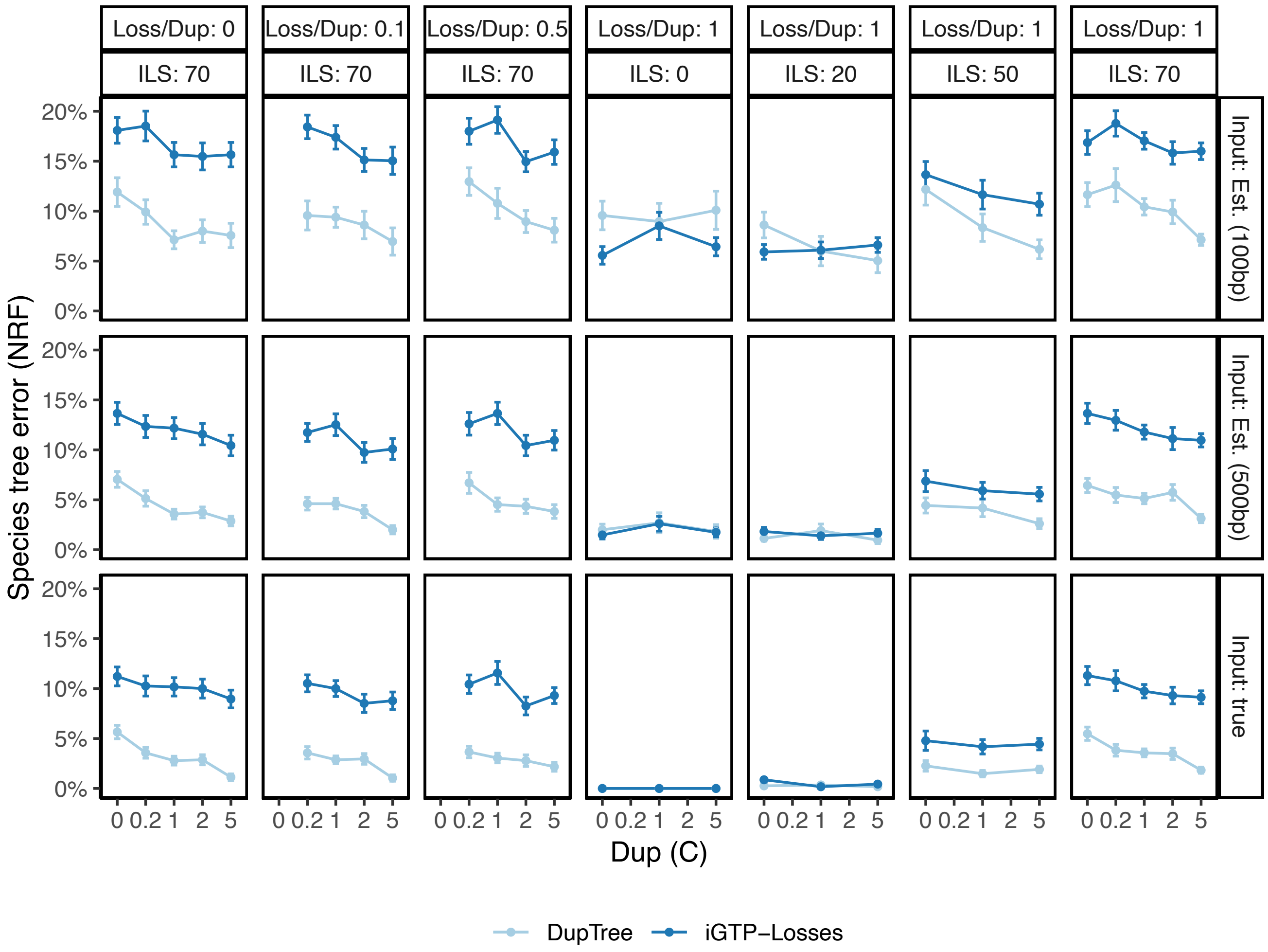

FIG. S7. Comparison of DupTree and iGTP-DupLoss methods on all the datasets with $n=25$ and $k=1000$. DupTree dominates iGTP-DupLoss in most conditions.

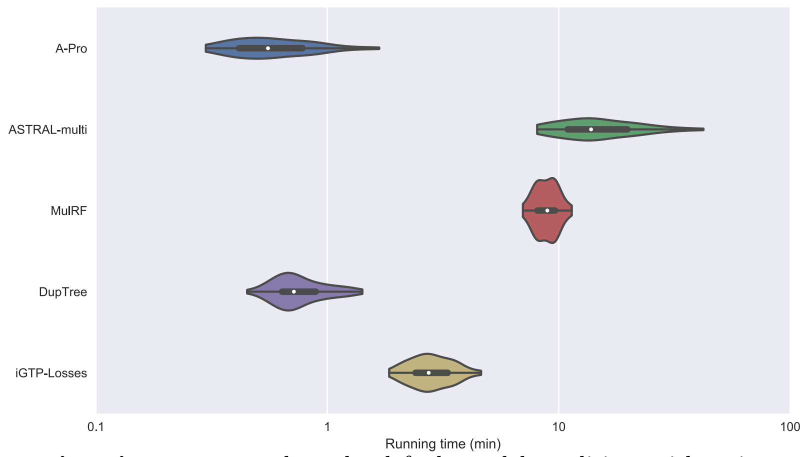

FIG. S8. Comparing running times, measured on the default model condition, with estimated gene trees (100bp). All methods are run in the single-threaded mode, on the same machine with Intel(R) Xeon(R) CPU E5-2670 0 @ 2.60GHz.

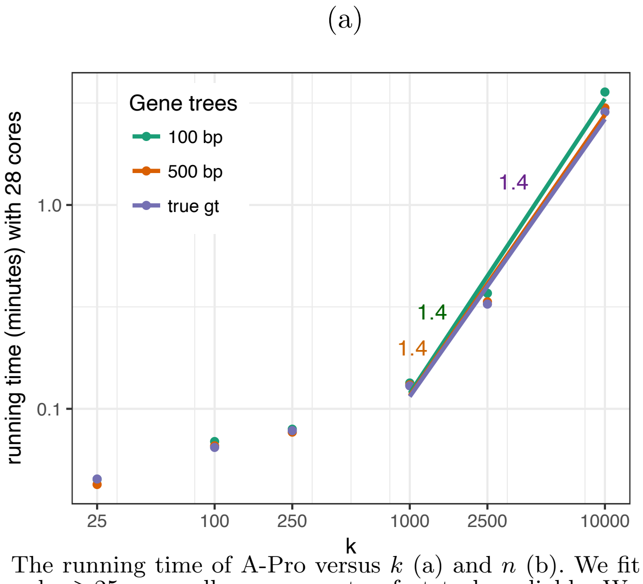

FIG. S9. The running time of A-Pro versus k (a) and n (b). We fit a line to the log-log plot of the running time only for $k \geq 1000$ and $n \geq 25$ as smaller runs are too fast to be reliable. We empirically estimate the A-Pro running time to grow roughly proportionally with $k^{3/2}$ and n^2.

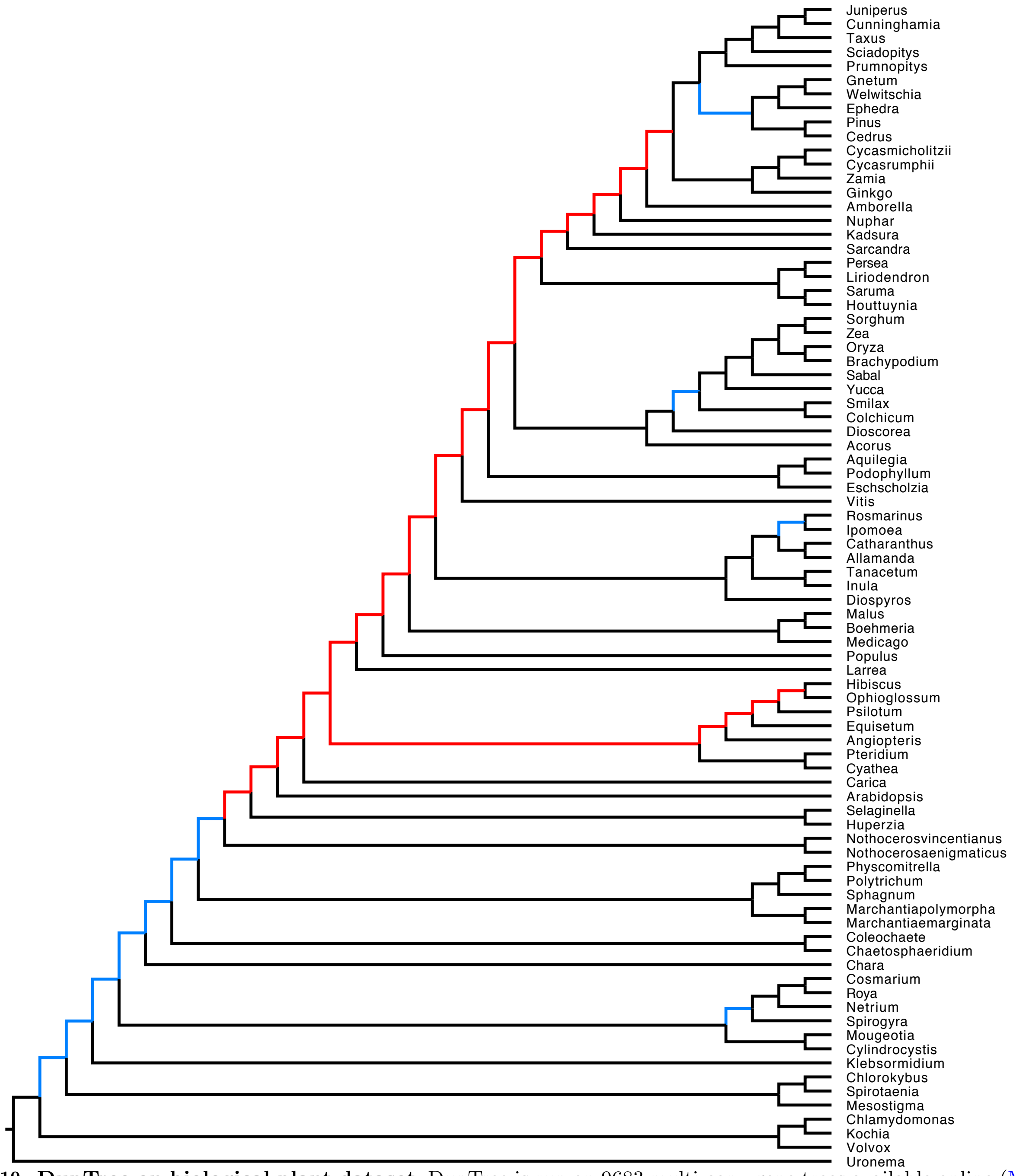

FIG. S10. DupTree on biological plant dataset. DupTree is run on 9683 multi-copy gene trees available online (Matasci *et al.*, 2014) for the plant dataset. Red: Branches that are obviously wrong, because these branches contradict basic biological categorization. Blue: Branches that contradict ASTRAL on single-copy genes that are not so obviously wrong.

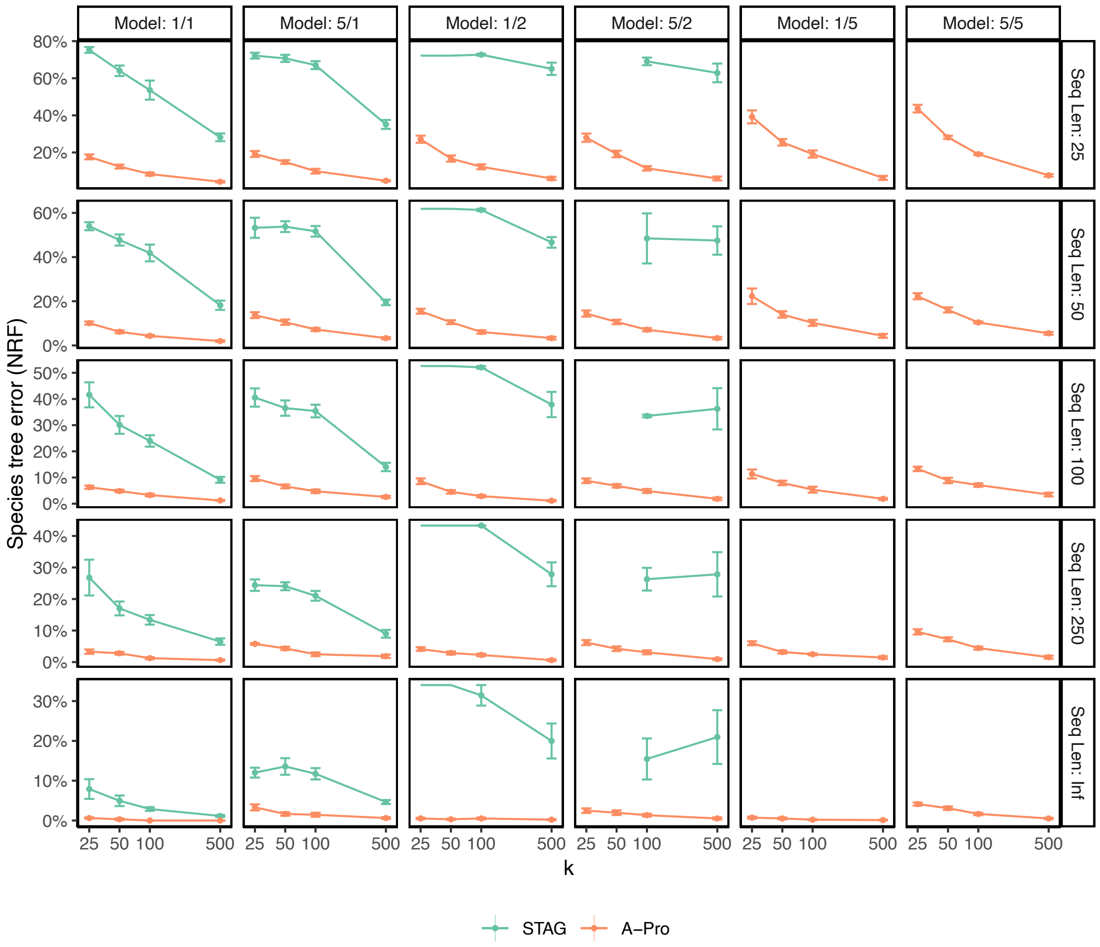

FIG. S11. Species tree error on S100 dataset. We compare the species tree error of the STAG method to A-Pro, showing mean and standard error over 10 replicates for each model condition, with varying numbers of genes (k) and sequence lengths (with Inf signifying true gene trees). Model conditions are labeled as a/b where a is the level of ILS (1 or 5) and b is the duplication/loss rate (1, 2, or 5). Cases with missing STAG results are due to STAG failing to run on those model conditions. Note that STAG infers a species tree from the input gene trees that have at least one leaf representing each species of interest; if none of the input gene trees satisfy this requirement, then STAG fails to return a tree.
